# Tempo and drivers of 3D eye size evolution in temperate butterflies

**DOI:** 10.1093/evlett/qrag001

**Published:** 2026-03-03

**Authors:** Sridhar Halali, Stephen A Hall, Lars B Pettersson, Romain Carrié, Paul Caplat, Emily Baird, Niklas Wahlberg

**Affiliations:** Biodiversity & Evolution Unit, Dept. of Biology, Lund University, Lund, Sweden; Division of Solid Mechanics, Lund University, Lund, Sweden; Biodiversity & Evolution Unit, Dept. of Biology, Lund University, Lund, Sweden; Centre for Environmental and Climate Science, Lund University, Lund, Sweden; Dynafor, National Research Institute for Agriculture, Food and Environment, INRAE, Toulouse University, Toulouse, France; School of Biological Sciences, Queen’s University Belfast, Belfast, United Kingdom; Department of Zoology, Stockholm University, Stockholm, Sweden; Biodiversity & Evolution Unit, Dept. of Biology, Lund University, Lund, Sweden

**Keywords:** long-term monitoring, microCT imaging, macroevolution, museum material, phenotypic evolution, sensory trait evolution

## Abstract

Sensory traits shape animal lifestyles due to the central role they play in retrieving and processing environmental information. However, being some of the most energetically expensive tissues to build and maintain, ecological demands often modulate investment in these organs. Evidence that ecology shapes the evolution of sensory traits is plenty, but is heavily biased towards vertebrates and has only recently begun to emerge in invertebrates. Here, we elucidate the macroevolution of a key sensory organ—eye size—using temperate butterflies as models. Using micro-CT X-ray imaging of pinned museum specimens, we quantified the eye size of 443 individuals comprising 59 species. Further, using 12 years of long-term monitoring data to quantify species habitat, we tested the hypothesis that forest-associated species, likely experiencing dimmer light conditions, should have larger eyes than those from open habitats. Our comparative analyses revealed tight allometric scaling between eye and wing size, and phylogeny alone explained 74% of eye size variation, with low heterogeneity in the evolutionary rates. Further, we found that habitat structure had no association with eye size. Overall, our findings indicate that allometry and shared ancestry, not ecology, shape the macroevolution of 3D eye size in temperate butterflies. We also demonstrate how non-invasive microCT imaging can be used on pinned museum specimens for studying phenotypic evolution on a macroevolutionary scale.

## Teaser

Insect sensory traits (e.g., eyes and antennae) can shape their lifestyles. However, sensory traits are among the most expensive tissues to build and maintain, and investment in them is often shaped by ecological factors. In our study, we used butterflies as a model system to investigate patterns and drivers of eye size evolution across 100 million years. We quantified eye size using 3D micro-CT X-ray imaging of pinned museum specimens. We find that species body size largely dictates eye size, and closely related species have similar eye sizes. In fact, species relatedness alone explains 74% of eye size variation. Intriguingly, habitat of a species (i.e., whether they are found more in forest or open habitats) did not influence eye size. Overall, we show that eye size evolution in temperate butterflies is strongly driven by body size and species relatedness, not ecological factors. We also demonstrate how non-invasive micro-CT technology can be used on museum specimens to study evolutionary patterns and processes across millions of years.

## Introduction

By retrieving and processing environmental information, sensory organs are essential for guiding animal behaviour, playing an important role in shaping animal lifestyles. However, sensory organs are among the most energetically expensive tissues to build and maintain (Niven & Laughlin, 2008). As such, the degree to which an animal invests in them is expected to be under strong selection and modulated according to specific ecological demands (Laughlin et al., 1998; Niven & Laughlin, 2008). There is plenty of evidence that ecological factors drive the evolution of sensory traits, at both micro- and macroevolutionary scales and across animals, in line with adaptive explanations. For example, nocturnal Lepidoptera have larger antennae than diurnal species (Lin et al., 2025), many fishes living in caves have completely lost their eyes (Krishnan & Rohner, 2017), and birds living in forests have larger eyes than those from open habitats (Ausprey et al., 2021). However, our knowledge of the macroevolution of sensory traits (e.g., eye size) is heavily based on vertebrates, with only a few recent studies emerging on invertebrates (Chong et al., 2024; Keesey et al., 2019; Scales & Butler, 2016; Wainwright et al., 2025).

Several factors can complicate studying sensory traits, especially on a macroevolutionary scale: (1) sensory traits generally show tight allometric scaling with body size (e.g., Ausprey, 2021; Chong et al., 2024) and such scaling could act as a constraint; (2) sensory traits (e.g., eye size) tend to have a strong phylogenetic signal (e.g., Ausprey, 2021), suggesting that shared ancestry could shape the evolutionary trajectory; (3) several studies do not find evidence for ecological factors shaping investment in sensory traits across animals (e.g., Chen et al., 2021). Thus, disentangling the relative roles of allometry, shared ancestry, and ecological factors is crucial to fully understand the evolution of sensory traits across species, a goal that can be achieved using a macroevolutionary framework.

The eye is a primary external sensory organ that is paramount for shaping most basic activities in visually guided animals. In both invertebrates and vertebrates, the light environment acts as a major selective force in determining eye size, anatomy, optics, and underlying molecular machinery (Caves et al., 2017; Sondhi et al., 2021; Warrant & Johnsen, 2013). For example, diurnal and nocturnal insects tend to show anatomically distinct apposition and superposition eyes, respectively, adapted for markedly different light environments (Land & Fernald, 1992; Land & Nilsson, 2012). While the light environment during the day and night is dramatically different, relatively subtle differences in the amount of light between habitats or at different depths in aquatic ecosystems can affect eye size and optics in both invertebrates and vertebrates (Ausprey et al., 2021; Scales & Butler, 2016; Terai et al., 2017; Wainwright et al., 2025). Although the anatomy of the insect compound eye is fundamentally different from camera-type eyes of vertebrates (see Land & Nilsson, 2012), the general principle of how selection operates on the eye surface area or the cornea is similar—the larger the eye surface area, the more light will be captured increasing sensitivity of the eye (Rutowski et al., 2009; Wright et al., 2024). However, many aspects of compound eye morphology, such as the facet size, facet number, and angle between the facets, ultimately determine the sensitivity and resolution (Land, 1997). Nevertheless, eye size can still indicate overall investment in the visual system, imposing significant energetic cost, and selection is likely to operate on this trait either increasing or decreasing the size depending on ecological demands (e.g., Beston et al., 2019; Moran et al., 2015; Tan et al., 2005).

Here, we elucidate the macroevolutionary dynamics of eye size using temperate butterflies as a model system. By generating 3D images of pinned museum specimens using micro-computed X-ray tomography (micro-CT), we quantified the 3D eye surface area of 443 individuals comprising 59 European butterfly species across major families spanning the evolutionary history of ∼100 million years. Using micro-CT is a major advance compared to previous studies on the evolution of sensory traits in insects, as: (1) few studies have used a macroevolutionary framework and many of those are restricted to a small taxonomic group (Keesey et al., 2019; Wainwright et al., 2025); (2) previous studies have mainly extrapolated eye surface area from linear measurements (e.g., Rutowski, 2000); and although using corneal spreads can provide accurate estimates of eye surface area (Wainwright et al., 2025; Wright et al., 2024), this method involves destructive specimen preperation. Next, using phylogenetic comparative methods, we investigate the allometry, tempo, and drivers, of eye size evolution. We further use 12 years of butterfly monitoring data from Sweden to quantify species habitat and test a hypothesis that forest-linked species, which would experience dimmer light conditions, should have relatively larger eyes (Ausprey, 2021; Ausprey et al., 2021; Wainwright et al., 2023). Overall, we provide first-hand insights into the macroevolutionary dynamics of eye size using temperate butterflies as models and demonstrate how high-throughput non-invasive X-ray micro-CT technology can be used on pinned museum specimens for studying phenotypic evolution.

## Materials and methods

### X-ray microCT scanning of museum specimens

We used pinned butterfly specimens from the Biological Museum at Lund University for micro-CT scanning without any pre-processing of the samples (i.e., no staining with heavy metals that is usually done for fresh samples and leaving specimens on their pins). The micro-CT scanning was carried out using an RX Solutions EasyTom150 (Chavanod, France) at the 4D Imaging Lab at Lund University. We scanned the butterfly heads using a polychromatic X-ray tube source (Hamamatsu L12161-07) with the source voltage set to 80kV and a current of 125 milli Amp using the small spot size setting (5 μm spot size). The source object distance was 56mm and the source detector distance was set to 894 mm. For each specimen, we acquired 992 projection images of 840 × 1456 pixels over 360° sample rotation using a Varian PaxScan 2520DX flat panel detector. The exposure time was set to 3 frames/s with an average of 2 frames per projection. 3D tomographic reconstruction was performed using the RX Solutions Xact software to yield 16-bit TIFF images with cubic voxels ranging from 6-9 μm resolution. Each sample was mounted with the metal pin as close to horizontal as possible, such that a clear view of the head could be obtained without artefacts from the pin, even accounting for cone-beam effects. We aimed to scan four males and four females of each species, but this was not always possible (see Table S1 for sample size). After excluding bad quality scans, we included 443 scans comprising 59 European butterflies for measuring eye surface area.

### Measuring the eye surface area and wing traits

**Figure 1:**
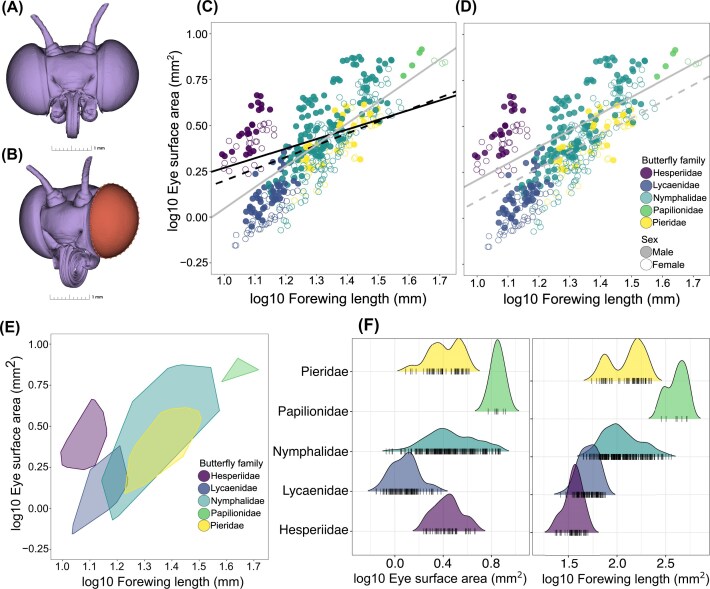
3D reconstructed head of a butterfly (Issoria lathonia, A) and the segmented corneal surface (highlighted in red, B) representing the 3D eye surface area. Allometry between 3D eye surface area and forewing length for all individuals (443 individuals, 59 species) is shown panel C and D. In panel C, the grey regression line represents the estimates from the ordinary least squares regression, and the solid and dashed regression lines represent estimates from PGLS with and without Hesperiidae, respectively. In panel D, solid and dashed PGLS regression lines are for male and female, respectively. Panel E shows the morphospace and F shows the combined distribution of the eye surface area and forewing length for all individuals including males and females. In all plots, points and polygons are colored according to the family (see inset in panel D), and filled and open circles in panels C and D represent males and females, respectively.

The 3D reconstructions, comprised of the 16-bit greyscale image stacks, were used for measuring the surface area of one of the eyes (Figure 1A, B, Figure S1). Knowing whether the right or left eye was measured is difficult as the order of image stacks can flip, thus affecting the symmetry. To check for any potential bias this may have caused, we measured the surface area (henceforth eye size) of both eyes on a subset of 20 randomly selected scans and found them to be highly similar (R^2^ = 0.99). We analysed all 3D scans in the open-source software 3D Slicer (Fedorov et al., 2012; Kikinis et al., 2013, https://www.slicer.org/) using additional functionalities from the *SlicerMorph* plugin (Rolfe et al., 2021). Additionally, we took 2D photographs of the same individuals and, using a custom macro in the Fiji software (Schindelin et al., 2015), measured forewing length and proxy forewing area (Figure S2). Both wing traits were highly correlated in our dataset (R^2^ = 0.98, Figure S3). We note that studies on butterfly eye size use different body size proxies, including hindleg femur/tibia length (Rutowski, 2000; Wright et al., 2024), inter-ocular distance (Wainwright et al., 2023), and thorax size (Moradinour et al., 2021). We use forewing length as this trait is often used as a body size proxy in butterflies (Swanson et al., 2016), is tightly correlated with diverse body size measures such as thorax size, pupal size, body length and body mass (García-Barros, 2025), and can be easily measured, especially when working with museum specimens. Details on processing 3D and 2D images are provided in the Supplementary Information.

### Repeatability of trait measurements

Since we manually measured both eye size (e.g., by manually placing a curve which demarcates the eye boundary) and wing traits (by manually placing the landmarks), and by two individuals (see Figures S1, S2), we measured repeatability for both traits as R^2^ values from the regressions. For eye size, we re-measured a subset of 20 randomly selected 3D images for: (1) intra-individual repeatability—original eye size vs. measurements carried out after 2-3 weeks; (2) inter-individual repeatability; (3) across software repeatability by measuring eye size in another popular 3D image analysis software Amira (Thermo Fisher Scientific, USA). We also quantified the repeatability of wing trait measurements by measuring them twice. The measurements were highly repeatable for both eye size and wing traits (R^2^ = 0.99, Figure S4).

### General software usage

Unless specified, we carried out all analyses in R ver 4.5.0 (R Core Team, 2025) and details of R packages used for specific analyses are provided when describing the analyses. Base R and *tidyverse* ver. 2.0.0 (Wickham et al., 2019) was used for general data handling, *ggplot2* ver. 3.5.2 (Wickham, 2016) and *ggridges* ver 0.5.6 (Wilke, 2024) for plotting non-phylogenetic figures, *ape* ver. 5.8.1 (Paradis & Schliep, 2019) for handling phylogenies and related data, *ggtree* ver 3.16.0 (Yu et al., 2017) for visualising phenotypic data on phylogenies, and *viridis* ver 0.6.5 (Garnier et al, 2024) for colour palette. Figure panels were made using Inkscape (https://inkscape.org/).

### Evolutionary allometry and the contribution of phylogeny in explaining eye size evolution

We used the published phylogeny of the European butterflies (Wiemers et al., 2020) for all comparative analyses and pruned it to match the number of species (n = 59) in the study. Unless specified, all trait data were log10 transformed before analysis. We carried out evolutionary allometry between eye size and forewing length by fitting several phylogenetic regressions with a phylogenetic covariance matrix as a random factor. First, we fitted a global regression (eye size ∼ forewing length) on the entire dataset (443 individuals of 59 species) to explore general allometric scaling. Next, we included Sex as an additive effect (eye size ∼ forewing length + sex) to test for sexual size dimorphism in the eye size. Visual inspection of the allometry indicated that the butterfly families occupy a unique morphospace (Figure 1E). We thus fitted two additional models, including only Family and both Family and Sex as additive effects. We avoided fitting a model that included three-way interactions between forewing length, Family and Sex as it became too complex, and our goal is to understand overarching allometric patterns rather than sex-specific scaling within families. The fit of the models was assessed using the Akaike Information Criteria (AIC) value.

The Hesperiidae family has superposition eyes (Horridge et al., 1972), unlike most diurnal butterflies, which have apposition eyes. Butterflies from the family Hedylidae (*Macrosoma sp*.) also have superposition eyes but are mainly nocturnal (Yack et al., 2007) and are not sampled in this study. As the sensitivity and resolution constraints on superposition eyes are different from apposition eyes (for details, see Land & Nilsson, 2012), this family acts as an ‘anatomical’ outlier. We therefore fitted a global regression model by removing Hesperiidae to test its effect on the estimates. Phylogenetic regressions were fitted using the *pGLMM* function in the *phyr* ver 1.1.0 R package (Ives et al., 2020).

Lastly, we quantified the contribution of shared ancestry by computing residual variation (R^2^) between the global phylogenetic to a non-phylogenetic (fitted using the *glm* function) regression using the *rr^2^* ver 1.1.1 package (Ives & Li, 2018).

### Extracting eye size residuals, phylogenetic signal, and fitting homogenous-rate evolutionary models

To account for allometric scaling between eye size and forewing length, we obtained relative eye size as residuals by fitting phylogenetic generalised least squares (PGLS) regression on species-averaged eye size and forewing length using the *gls* function from the *nlme ver* 3.1.168 package (Pinheiro & Bates, 2000, Pinheiro et al., 2025). Initially, we fitted separate PGLS for males and females, however, their eye size residuals followed the same pattern across species (Figure S5). Similarly, we fitted separate PGLS by removing Hesperiidae which are anatomical outliers and occupy a unique position in the morphospace (Figure 1E) to its test effect on the residuals. We found that except for a few species, the patterns in the residuals were broadly similar (Figure S6). Thus, unless specified, we carried out further comparative analyses on pooled species-averaged traits including all 59 species. PGLS was supplied with the Brownian Motion, Ornstein-Uhlenbeck and Pagel’s λ correlation structures, along with a non-phylogenetic model (Pagel’s λ=0). Models with the Brownian Motion and Pagel’s lambda correlation structure had a similar fit (Table S2) and the likelihood ratio test was non-significant (P = 0.24). Thus, we used residuals from the Brownian Motion model for quantifying phylogenetic signal and the fit of evolutionary models (see below). Using residuals as a response variable, especially when fitting multiple regression models, can be problematic (Freckleton, 2002, 2009). We therefore use eye size residuals only in cases where it is not possible to include forewing length as a covariate (see below).

Eye size residuals were used to calculate phylogenetic signal (as Pagel’s λ) and for fitting models of continuous trait evolution. We quantified Pagel’s λ using the *phylo.sig* function in the *phytools* ver 2.4.4 package (Revell, 2012, 2024). Pagel’s λ ranges from 0 to 1, where λ = 0 indicates no effect of shared ancestry on trait evolution, and λ=1 indicates a strong phylogenetic signal, consistent with a Brownian Motion model of evolution. We further fitted several homogenous-rate evolutionary models (Brownian Motion, Ornstein-Uhlenbeck, Early Burst, Brownian Motion with a trend and a non-phylogenetic white noise model) using the *fitContinuous* function in the *geiger* ver 2.0.11 package (Harmon et al., 2008; Pennell et al., 2014). Model fits were assessed using the AICc score and AIC weights. We tested for the effect of phylogenetic uncertainty on phylogenetic signal using the *sensiPhy ver* 0.8.5 package (Paterno et al., 2018) and fit of continuous trait evolution models by replicating analyses on 500 posterior trees.

### Fitting heterogeneous-rate evolutionary models

Variation in the rate of trait evolution through time or across lineages is pervasive (Chira & Thomas, 2016). To account for rate heterogeneity in evolutionary rates across clades, we first used the Bayesian variable-rates regression model (Venditti et al., 2011). The model first computes the expected trait values based on the homogeneous-rate Brownian Motion model (with global background rate) and compares them to the observed trait values. If the observed trait values do not conform to the background rate, the model will then either stretch or compress the branches or clades such that trait evolution conforms to the Brownian Motion model. The rate scalar (*r*) determines the magnitude at which the branches/clades are stretched or compressed and can indicate branches/clades evolving at exceptional rates: *r* > 1 or 0 ≤ *r < 1* indicates the evolutionary rate is higher or lower than the background rate, respectively, while *r* = 1 indicates the rate is same as the background Brownian rate (Baker et al., 2016).

The variable-rates model uses the reversible-jump Markov Chain Monte Carlo (MCMC) approach and is implemented in the software *BayesTraits* ver 4.1.3 (Meade & Pagel, 2024). Species-averaged trait data were supplied in the form of a regression (mean eye size ∼ mean forewing length), and the model then returns a posterior distribution of regression coefficients, branch-specific rate scalars (*r*), background Brownian rate and Pagel’s λ value. Additionally, we also fitted the homogenous-rate model where rates do not vary across clades. We ran three independent MCMC chains for 110 000 000 iterations with a burn-in of 10 000 000 and sampling every 10 000 iterations thereafter for both models. Following previous studies (Baker & Venditti, 2019; Furness et al., 2022), the gamma prior (α=1.1, β rescaled to give a median of 1) was provided for the scalar parameter, a uniform prior ranging between −100 to 100 for regression coefficients, and a uniform prior ranging between 0 to 1 for Pagel’s λ. MCMC diagnostics were assessed visually by inspecting trace plots and calculating effective sample size (>1000 for all estimated parameters) and Gelman-Rubin metric (value of 1–1.2 is expected for converged chains) using the *coda* ver 0.19.4.1 package (Plummer et al., 2006). Examination of trace plots suggested the likelihood value for the homogenous-rate model was more constrained and remained near an upper bound, although the effective sample size was high and the Gelman-Rubin metric was 1. Convergence diagnostics are reported in Supplementary Information (see Table S3, Figure S7). We calculated the Bayes Factor to test if the variable-rate model fits better than the homogenous-rate model using the formula: *2(log marginal likelihood of complex model—log marginal likelihood of simple model)* (Table S4). The complex and simple models here are variable- and homogeneous-rate models, respectively. The value of the Bayes Factor > 2 suggests better support for the complex or the variable rates model (Meade & Pagel, 2024).

Additionally, we fitted a recent penalised-likelihood based variable-rates model (Revell, 2021) to explore rate heterogeneity in eye size residuals (as including a covariate was not possible). The model can be supplied with different values of λ (this is not the same as the Pagel’s λ) where lower values allow more rapid changes in the evolutionary rates, while higher value impose higher penalty and the rates are relatively conserved. Determining optimal λ value apriori is not possible, but one can run models with different λ values and check if certain clades always have higher or lower rates (Revell, 2021). We fitted the model using four different λ values (0.1, 1, 10, 50) (see Revell, 2021) and then visually inspected for clades consistently showing higher or lower rates. The model was fitted using the *multirateBM* function in *phytools*.

### Quantifying species habitat

We used species habitat (affinity towards closed or open habitats) as a proxy for the light environment—species in more forested habitats will experience relatively dimmer light conditions than in open habitats. For quantifying species habitat, we carried out spatial analyses using the aggregated long-term monitoring data (from 2010-2021) from the Swedish Butterfly Monitoring Scheme (SEBMS, Pettersson, 2025) and the Copernicus tree cover density raster (10 × 10m^2^ grid size) for Sweden (workflow shown in Figure 3). SEBMS conducts systematic butterfly surveys every year on fixed transects all over Sweden (Figure 3B). Such systematic survey data should have lower observation bias which is typically observed in citizen science databases (Beck et al., 2014). Each transect is divided into several segments depending on the transect length (Figure 3C) and is generally composed of a similar habitat. Since our goal was to quantify broad species habitat association, we used tree cover density as a proxy of habitat structure for each segment on the transect and assigned this value to all species recorded on the segment. Thus, we obtained the distribution of habitat association for each species, and we used the median value of this distribution (Figure 3D, 3E) for further analyses. In total, we quantified habitat across 1427 unique transect segments on which 19 889 individuals of 57 species were recorded.

We used the tree cover density raster and extracted the values for all grids where the raster intersected with butterfly monitoring transects using the *exactextractr* ver 0.10.0 package (Baston, 2023). We set the buffer of 10m on each side of each transect and then calculated the weighted mean of canopy cover for each segment of the transect by weighting with the coverage fraction. Coverage fraction is the degree of overlap between the buffered transects, with a value of 1 indicating 100% overlap and 0 indicating no overlap. We excluded points with < 0.002 coverage fraction.

### Testing the effect of habitat on eye size

We tested the association between eye size and habitat (using median tree cover density as a proxy obtained from spatial analyses, Figure 3D) across species by fitting PGLS (same steps as described above) with species-averaged eye size as a response, forewing length as a covariate, and median tree cover density as a predictor. We fitted an additional model to test how absolute eye size (i.e., by removing forewing length) and tree cover density are associated. Both covariate and predictor were standardised (mean = 0, SD = 1) before fitting the models. Additionally, using the aggregated data across years (since we do not investigate the putative temporal changes in the species assemblages and traits), we calculated community-weighted means for species-averaged eye size, eye size residuals and species-averaged forewing length. Community-weighted means were calculated by averaging the trait values of recorded species at each segment of the transect weighted by their observed average abundance across years. These weighted means were regressed against the median tree cover density values of the segment separately using linear models. Finally, we fitted all above described models only for the Nymphalidae family, given the moderate sample size (32 species) and species representative of forest- and open-like habitats from different clades.

## Results

### Tight allometric scaling and family-specific morphospace

Global phylogenetic regression using *pGLMM* on the entire data set revealed tight scaling between 3D eye surface area (henceforth eye size) and forewing length (estimate = 0.52, P < 0.001; AIC= −916.6, Figure 1C). Excluding the family Hesperiidae from the global regression resulted in a slightly steeper slope (estimate = 0.65, P < 0.001, Figure 1C). Compared to the global model, including Sex as both an additive or interactive fixed effect with the covariate (forewing length) greatly improved the AIC score. For example, including Sex as an additive effect with forewing length as a covariate further suggested that males generally have larger eyes than females relative to wing length (estimate of Sex_male_ = 0.11, p < 0.001, AIC = −1342.4, Figure 1D). When Sex was included as an interactive effect, females had slightly steeper slope than males (estimate of Forewing length * Sex_male_ = −0.06, p = 0.0311, AIC= −1344.9, Table S5, Figure S8). Models that included only Family as an additive effect had a worse fit (AIC= −914.8) than the global model. Thus, we do not place much emphasis on family-level patterns (but see Figure S8 for allometry). But including both Family and Sex as an additive effect greatly increased the model fit (AIC = −1342), which is similar to the model where Sex was included as an additive effect. Briefly, this model suggested that, in addition to males generally having larger eyes than females, butterflies from the families Lycaenidae and Pieridae had relatively smaller eyes than family Hesperiidae (Table S5). Parameter estimates of all allometric models are provided in Table S5.

**Figure 2:**
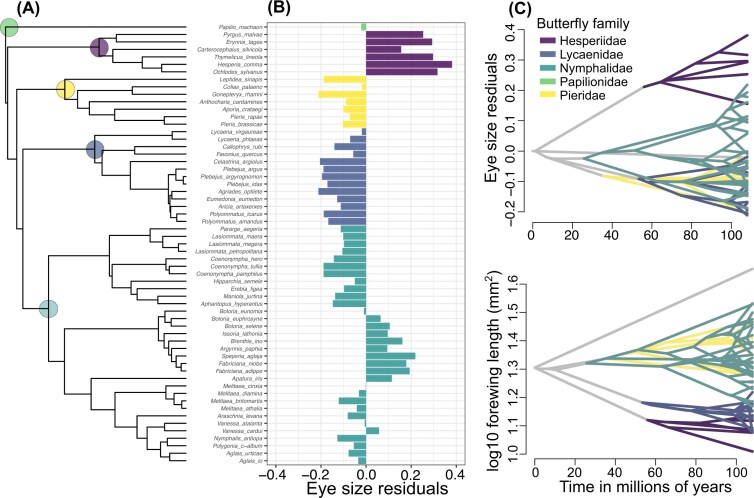
The phylogeny of 59 temperate butterflies (A) included in the study with families are highlighted using solid circles at the nodes. In panel B, eye size residuals were obtained from phylogenetic regression (log10 eye size∼log10 forewing length) using species-averaged trait values. In panel C, eye size residuals and forewing length are projected onto a phylogeny following the Brownian Motion model of evolution. In all panels, colors represent butterfly families (see inset in panel C).

Visualizing the allometry indicated that families occupy unique morphospace (Figure 1E). For example, species in the family Lycaenidae are generally smaller (hence smaller forewing length) and have smaller eyes. While species in the family Nymphalidae show large variation in forewing length, and thus eye size is more spread out in the morphospace (Figure 1E, F). The exception was the family Hesperiidae, which has a much larger relative eye size (Figure 2A). This pattern was also mimicked in the phenogram of eye size residuals, which showed that Hesperiidae and a clade of fritillary butterflies had the largest eyes for their body size (Figure 2C).

Finally, calculating the residual variation by comparing the phylogenetic and non-phylogenetic regression (eye size∼forewing length) suggested that phylogeny accounted for 74% of the variation in eye size evolution.

### Eye size residuals have a strong phylogenetic signal and follow the Brownian Motion model of evolution

The phylogenetic signal (Pagels λ) in eye size residuals was high (λ=0.98, Figure 2, see Figure S9 for the likelihood profile) and remained similarly high across 500 posterior trees (Figure S9). Further, the Brownian Motion model had a better fit among other models and was the case across 500 posterior trees (Table S6, Figure S10). However, we interpret this result with caution as model convergence was low for Ornstein-Uhlenbeck, Brownian Motion with trend and Early Burst models (Table S6).

### Rate heterogeneity in eye size evolution is low and is family-specific

The variable-rate model did not have a better fit than that of the homogeneous-rate model (average Bayes Factor across three runs = 0.23, Table S4). Almost all median rate scalars (*r*) were 1, suggesting that the residual variation in the Brownian rate and background Brownian rate were similar, except for the Hesperiidae family with median *r* > 10 (Figure S11). This is apparent in the consensus tree obtained from the variable-rates model where the entire Hesperiidae clade is stretched out, showing a higher evolutionary rate (Figure S11). Similarly, the clade of fritillary butterflies (especially from genera *Fabriciana* and *Speyeria*) also have relatively large eyes are only slightly stretched out (Figure S11). The patterns were congruent with the penalized-likelihood based variable-rates method showing that Hesperiidae and the clade of Fritillary butterflies had relatively higher rates, and also that the Satyrinae butterflies had relatively lower rates (Figure S12).

### Habitat has a weak negative effect on eye size, but it depends on the method

**Figure 3:**
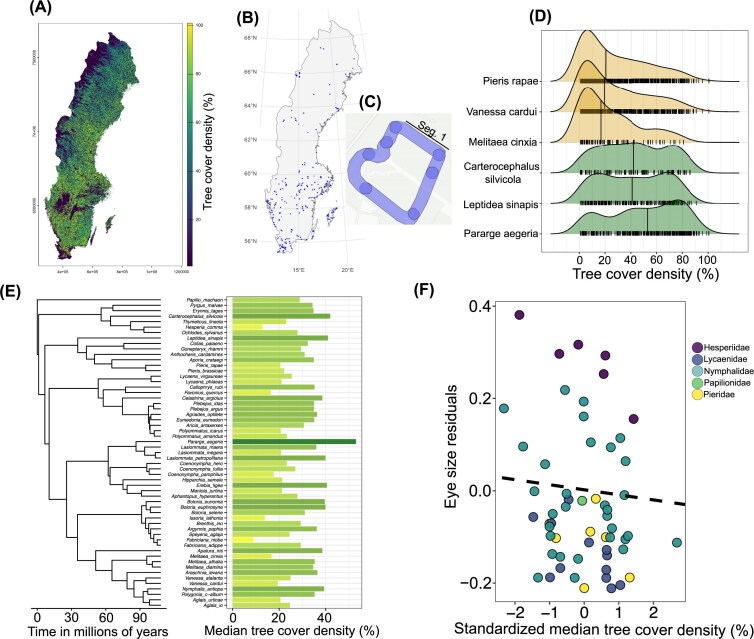
Workflow of the spatial analyses to quantify species habitat preference and test the association between eye size and habitat. Copernicus tree cover density (in %) raster (A) and the transects (B) used for monitoring butterflies by the Swedish Butterfly Biomonitoring Scheme across Sweden. Each transect is composed of different segments (C, intersections represent the start and end of the segment), depending on the length of the transect. Aggregated butterfly presence data (across 12 years, from 2010-2021) for each segment were used, and the habitat was quantified at the segment level such that all butterflies recorded at a particular segment would be assigned the same value of habitat metric (i.e., % tree cover density). But note that not all segments have the sampling effort of 12 years. From this, a distribution (D) for each species was obtained, and the median tree cover density value (black vertical line) was used as a predictor in phylogenetic regressions. Median tree cover density for 57 species is shown in (E). The relationship between eye size residuals and median tree cover density (F) was tested using PGLS (estimate= −0.01, P = 0.09). Colors in regression plot (F) represent butterfly families. Note that core PGLS models were fitted including forewing length as a covariate to control for allometric scaling (see Methods).

PGLS indicated habitat did not influence either relative (estimate= −0.01, P = 0.08, Figure 3F, Tables S7, S8) or absolute (estimate = −0.001, P = 0.90, Figure S13, Tables S7, S8) eye size. However, regressions between community-weighted means of relative (estimate = −0.0003, P = 0.004) or absolute eye size (estimate = −0.0003, P < 0.001) and tree cover density showed a very weak negative association, with large confidence intervals around estimates (Figure S14, Table S9). The association between community-weighted forewing length and canopy cover was non-significant (Figure S14, Table S9). Finally, carrying out PGLS and regressions on community-weighted means only for the family Nymphalidae indicated no effect of habitat on relative and absolute eye size (Figure S14, Tables S10-12, S11, S12).

## Discussion

Sensory organs are some of the most energetically expensive tissues to build and maintain, and their investment is often strongly modulated by ecological factors. Here, we elucidate macroevolutionary dynamics and drivers of eye size evolution in temperate butterflies by leveraging museum collections and microCT technology.

Evolutionary allometry indicated a tight scaling between eye size and forewing length (a body size proxy) and that butterfly families occupy a unique position in the morphospace. This pattern arises because the forewing length, or body size in general, tends to be family-specific in butterflies. For example, Papilionidae are often large, most Pieridae are medium-sized, and species in the Hesperiidae and Lycaenidae are generally much smaller than species from other families, while Nymphalidae show a high degree of variation in forewing lengths across species (Figure 1F). Although our study has limited sampling, the family-specific pattern of body size holds across European butterflies (Figure S15, data from Middleton-Welling et al. (2020)). The family Hesperiidae stands out in the morphospace as they have much larger eyes to their relatively small forewing lengths compared to the same-sized butterflies from other families, for example, Lycaenidae (Figures 1, 2). This is explained by their superposition eyes with optics designed to optimise light capture in dark environments (e.g., nocturnal moths typically have superposition eyes) despite being diurnal, while other diurnal butterflies mainly have apposition eyes (Horridge et al., 1972). Interestingly, butterflies from the Hedyliadae family, which are known to be nocturnal, have superposition eyes (Yack et al., 2007). For a given size, superposition eyes are more light sensitive than apposition eyes (Land & Nilsson, 2012). By having superposition eyes that are relatively larger than butterflies from other families with similar body sizes, Hesperiidae butterflies would have a far greater light sensitivity and may facilitate their typical fast flight, allowing them to have fast motion vision. Thus, in a sense, Hesperiidae are ‘anatomical’ outliers within the diurnal lineage of butterflies. Removal of Hesperiidae from allometry, as expected, increased the slope by a small magnitude (Figure 1A).

The allometry also indicated that males generally have larger eyes than females relative to wing length, which is often the case in butterflies and insects (Rutowski, 2000; Stanger-Hall et al., 2018). It is hypothesised that larger eyes (or greater visual sensitivity) in male butterflies might aid in spotting rival males, mates or predators (Bergman et al., 2021; Rutowski, 2000). We posit another mutually non-exclusive hypothesis that females are faced with a larger energetic trade-off between investment in sensory and reproductive organs than males, leading to smaller sensory tissues such as eye size.

Eye size residuals had a strong phylogenetic signal, and phylogeny explained 74% variation in the eye size evolution. This pattern corroborates the findings of other studies. For example, along with tight allometric scaling between eye size and body size, Ausprey (2021) and Ausprey (2024) found a high phylogenetic signal (Pagel’s λ ≥ 0.9) for relative eye size, and that phylogeny explained > 60% variation in eye size in birds. Such a strong phylogenetic signal in the eye size residuals likely also explains the better fit for the Brownian Motion model of evolution. However, we interpret this result with caution as the fit of other models (i.e., Brownian-Motion with trend, Ornstein-Uhlenbeck, Early Burst) had poor convergence (see Table S6). Furthermore, the Bayesian variable-rates method to model rate heterogeneity had the similar fit as that of the homogenous-rate Brownian Motion model and failed to detect clades or branches with exceptional rates, except for Hesperiidae and a clade of Fritillary butterflies which had relatively higher rates (Figure S11). Similar trend was found using the penalized-likelhood based variable-rates method (Figure S12). Such a strong phylogenetic signal, conformity to the Brownian Motion model and low heterogeneity in evolutionary rates is striking, especially when sensory organs are expected to evolve under selective regimes such as light environment (Ausprey, 2021; Ausprey et al., 2021; Wainwright et al., 2025). One possible reason is that since eye size is allometrically dependent on body size, and since body sizes are family-specific, the evolution of eye size is constrained at the family level. In butterflies, major families diverged ∼60-90 million years ago (Chazot et al., 2019), suggesting that eye size and body size evolved deep in time. In sum, the best fit to the Brownian Motion model does not necessarily imply an absence of selection, rather, small adaptive shifts, especially those occurring on shallow evolutionary scales, will likely be masked by phylogenetic constraints and difficult to detect. For instance, a study on tropical Ithomiini butterflies with an evolutionary history of roughly 26 million years and a sample size similar to our study, multiple-optima adaptive Ornstein-Uhlenbeck models best explained the evolution of sensory traits (Wainwright et al., 2025).

Habitat structure influencing light environment is one of the major drivers of eye size evolution in diurnal lineages of animals. For example, butterflies and birds living in forested or close canopy habitats (=dim light) tend to have larger eyes than those living in open habitats (=bright light) (Ausprey, 2021; Ausprey et al., 2021; Wainwright et al., 2023; Wright et al., 2024). The test of this hypothesis in insects on a large macroevolutionary scale is, however, lacking (but see Wainwright et al., 2025). Here, we tested for the association between the light habitat association of a species (quantified as tree cover density) and both absolute and relative eye size using both phylogenetically controlled regressions and community-weighted means. Intriguingly, we found no association between habitat and relative and absolute eye size and a weak negative effect when community-weighted means were used. In fact, species in the clade of Fritillary butterflies included in our study which have the largest relative eye size along with Hesperiidae (Figure 2), many of those species occur in open habitats (Figure 3). The lack of relationship between eye size and light habitat or other factors that affect light environment (e.g., water depth) has also been found, for example, in butterflies (Seymoure et al., 2015), bumblebees (Bartholomée et al., 2023; Tichit et al., 2024), fishes (Yoder et al., 2022) and frogs (Chen et al., 2021; Huang et al., 2019). Taken together, our results show that the evolution of eye size is largely explained by allometry and shared ancestry, at least when examined at such a deep and broad macroevolutionary scale.

While eye size to some extent can respond to light conditions (or other ecological factors) in butterflies (Wainwright et al., 2025; Wright et al., 2024), different components of eye morphology, and not the eye surface *per se*, determine the sensitivity and resolution. Not being able to quantify detailed eye morphology is an important caveat of our study, limiting it from fully elucidating how ecology shapes visual adaptation. In the apposition compound eye type (which is the eye type of all butterflies tested here except for the Hesperiidae), each facet is one light-capturing optical unit (Land, 1997; Land & Nilsson, 2012). The number of facets and their distribution across the eye, facet diameter, the angle between the facets (i.e., interommatidial angle), and the eye curvature determine the sensitivity and resolution. For example, increasing the facet diameter *(D)*, which increases the interommatidial angle *(ΔΦ)*, allows greater capture of the light, increasing eye sensitivity (Land, 1997; Rutowski, 2000). In contrast, increasing the number of facets results in a decrease of the interommatidial angle, increasing resolution. The eye parameter (calculated as: *D×ΔΦ*) reflects the trade-off between eye sensitivity and resolution. For example, in bumblebees, species associated with dim light environments have a higher eye parameter (i.e., larger *D* and *ΔΦ)* than those associated with bright light environments, but there was no correlation with eye surface area (Bartholomée et al., 2023; Tichit et al., 2024). This relationship gets even further complicated when eye curvature enters the equation. For example, flatter eyes can have fewer larger facets increasing both sensitivity and resolution, but the same facets on a curved eye would maintain sensitivity at the expense of lower resolution due to an increase in the inter-ommatidial angle (Rutowski, 2000). To deal with the sensitivity-resolution trade-off, species often show regional differences in density of facets and facet diameter across an eye (Rutowski et al., 2009). Thus, in principle, even if the eye size remains similar across species irrespective of the habitat (as seen in our data), selection can operate and optimise different aspects of eye morphology. In the context of our study, even if a forest and open habitat species may have similar eye size, it is possible that eyes may differ in facet number, facet diameter or eye curvature, which ultimately determine how a butterfly or an insect would see its surroundings. Future studies using high-resolution scans (usually < 3μm) on fresh tissues with intact internal anatomy, such as the orientation of cones, would provide important insights into how ecology shapes visual adaptation.

In summary, we have leveraged high-throughput X-ray micro-CT imaging of museum specimens and long-term biomonitoring data to quantify 3D eye size and species habitat of European butterflies, respectively. Using a range of phylogenetic comparative methods, we show that allometry and shared ancestry, and not ecology, shape the evolution of eye size. Future studies on the evolution of eye morphology and anatomy on a macroevolutionary scale will provide holistic insights into how ecology shapes visual adaptations in invertebrates.

## Supplementary Material

qrag001_Supplemental_File

## Data Availability

All the data, intermediate and log files, scripts, and 3D models of eye surface area, are deposited on Zenodo: https://doi.org/10.5281/zenodo.18144261.
