# Supplementary material for: Tempo and drivers of 3D eye size evolution in temperate butterflies
Source: Evol Lett. 2026 Mar 3;10(2):195–206. doi: 10.1093/evlett/qrag001 (PMC13043916; doi:10.1093/evlett/qrag001)
Supplement: qrag001_Supplemental_File [file qrag001_supplemental_file.pdf]

SUPPLEMENTARY INFORMATION

Title: Tempo and drivers of 3D eye size evolution in temperate butterflies

Authors: Sridhar Halali, Stephen A. Hall, Lars B. Pettersson, Romain Carré, Paul Caplat, Emily Baird, Niklas Wahlberg

Table S1: Number of individuals per species and sex for which eye size and wing traits were measured.

Family	Genus	Species	Female	Male	Total count
Hesperiidae	Carterocephalus	silvicola	4	4	8
Hesperiidae	Erynnis	tages	4	4	8
Hesperiidae	Hesperia	comma	4	4	8
Hesperiidae	Ochlodes	sylvanus	4	4	8
Hesperiidae	Pyrgus	malvae	4	3	7
Hesperiidae	Thymelicus	lineola	4	4	8
Lycaenidae	Agriades	optilete	3	3	6
Lycaenidae	Aricia	artaxerxes	3	4	7
Lycaenidae	Callophrys	rubi	4	4	8
Lycaenidae	Celastrina	argiolus	3	4	7
Lycaenidae	Eumedonia	eumedon	3	3	6
Lycaenidae	Favonius	quercus	4	4	8
Lycaenidae	Lycaena	phlaeas	2	4	6
Lycaenidae	Lycaena	virgaureae	4	4	8
Lycaenidae	Plebejus	argus	4	4	8
Lycaenidae	Plebejus	argyrognomon	4	4	8
Lycaenidae	Plebejus	idas	4	4	8
Lycaenidae	Polyommatus	amandus	4	3	7
Lycaenidae	Polyommatus	icarus	4	4	8
Nymphalidae	Aglais	io	4	3	7
Nymphalidae	Aglais	urticae	4	2	6
Nymphalidae	Apatura	iris	1	4	5
Nymphalidae	Aphantopus	hyperantus	4	4	8
Nymphalidae	Araschnia	levana	4	2	6
Nymphalidae	Argynnis	paphia	4	4	8
Nymphalidae	Boloria	eunomia	4	4	8
Nymphalidae	Boloria	euphrosyne	4	4	8
Nymphalidae	Boloria	selene	4	4	8
Nymphalidae	Brenthis	ino	4	4	8
Nymphalidae	Coenonympha	hero	2	4	6
Nymphalidae	Coenonympha	pamphilus	3	4	7
Nymphalidae	Coenonympha	tullia	4	4	8
Nymphalidae	Erebia	ligea	3	4	7
Nymphalidae	Fabriciana	adippe	4	4	8
Nymphalidae	Fabriciana	niobe	3	4	7
Nymphalidae	Hipparchia	semele	4	4	8
Nymphalidae	Issoria	lathonia	4	4	8
Nymphalidae	Lasiommata	maera	4	4	8
Nymphalidae	Lasiommata	megera	4	3	7
Nymphalidae	Lasiommata	petropolitana	4	3	7
Nymphalidae	Maniola	jurtina	4	4	8
Nymphalidae	Melitaea	athalia	4	4	8
Nymphalidae	Melitaea	britomartis	4	4	8
Nymphalidae	Melitaea	cinxia	4	4	8
Nymphalidae	Melitaea	diamina	4	4	8
Nymphalidae	Nymphalis	antiopa	4	4	8

Nymphalidae	Pararge	aegeria	3	4	7
Nymphalidae	Polygonia	c-album	4	4	8
Nymphalidae	Speyeria	aglaja	3	4	7
Nymphalidae	Vanessa	atalanta	4	4	8
Nymphalidae	Vanessa	cardui	4	4	8
Papilionidae	Papilio	machaon	3	4	7
Pieridae	Anthocharis	cardamines	4	4	8
Pieridae	Aporia	crataegi	3	5	8
Pieridae	Colias	palaeno	4	4	8
Pieridae	Gonepteryx	rhamni	4	3	7
Pieridae	Leptidea	sinapis	4	4	8
Pieridae	Pieris	brassicae	4	4	8
Pieridae	Pieris	rapae	4	4	8

Table S2: Fit of the PGLS model (eye size ~ forewing length) supplied with different correlation structures. The models were fitted on the pooled species-averaged traits and were log10 transformed before fitting the models. Residuals from the best fit model were used for downstream evolutionary analyses. Note that the fit of the models with Brownian Motion and Pagel's lambda correlation structures are similar and the likelihood ratio test was non-significant. Thus, the residuals from the model with the Brownian Motion correlation structure was used for downstream evolutionary analyses.

Correlation structure	Log likelihood	No. of parameters	AIC
Brownian Motion	71.3046	3	-136.6091
Ornstein-Uhlenbeck	28.4658	4	-48.9316
Pagel's lambda	71.9933	4	-135.9867
OLS (Pagel's lambda=0)	28.4658	3	-50.9316

Table S3: Combined effective sample size and Gelman-Rubin estimates for the three independent runs of the Bayesian variable-rates and homogenous-rates model.

Model	Parameters	Effective sample size	Gelman-Rubin point est.	Gelman-Rubin upper CI
Variable-rates	Likelihood	29051	1	1
Variable-rates	Alpha	30131	1	1
Variable-rates	Beta	30306	0.99	1
Variable-rates	Variance	29605	1	1.09
Variable-rates	R^2	29470	1	1
Variable-rates	Beta std. error	29656	1	1
Variable-rates	Lambda	30857	1	1
Homogenous-rates	Likelihood	27736	0.99	0.99
Homogenous-rates	Alpha	30374	1	1
Homogenous-rates	Beta	30374	1	1
Homogenous-rates	Variance	29910	0.99	0.99
Homogenous-rates	R^2	27965	1	1
Homogenous-rates	Beta std. error	29756	1	1
Homogenous-rates	Lambda	30000	0.99	0.99

Table S4: Marginal likelihood values and Bayes Factor for three independent runs of variable-rates and homogenous-rates model. Bayes factor was calculated as: 2(marginal likelihood of complex model – marginal likelihood of simple model). Complex and simple models here are variable-rates and homogenous-rate models, respectively.

Runs	Marginal likelihood for variable-rates model	Marginal likelihood for homogenous-rates model	Bayes Factor
1	62.51	61.79	1.44
2	62.48	62.67	-0.38
3	62.41	62.59	-0.36
			Average BF: 0.23

Table S5: Estimates from the phylogenetic regressions fitted on the entire data (n=443 individuals comprising 59 species) using *pGLMM* function (from *phyr* R package). The specific structure of the fitted models are highlighted in the specific rows. All data was log10 transformed before fitting the models.

Model terms	Coefficient	Std.Error	Z Score	P value
Global model (Eye size ~ Forewing length)				
Intercept	-0.2559	0.1483	-1.7255	0.0844
Forewing length	0.5245	0.1020	5.1443	<0.001
Global model without Hesperiidae (Eye size ~ Forewing length)				
Intercept	-0.4482	0.1589	-2.8209	0.0048
Forewing length	0.6466	0.1052	6.1483	<0.001
Sex as an additive effect (Eye size ~ Forewing length + Sex)				
Intercept	-0.8099	0.1079	-7.5044	<0.001
Forewing length	0.9032	0.0717	12.5998	<0.001
Sex-Male	0.1148	0.0042	27.0360	<0.001
*Sex as an interactive effect (Eye size ~ Forewing length * Sex)*				
Intercept	-0.7919	0.1088	-7.2771	<0.001
Forewing length	0.8903	0.0720	12.3643	<0.001
Sex-Male	0.1950	0.0375	5.1968	<0.001
Forewing length:Sex-Male	-0.0632	0.0293	-2.1552	0.0311
Family as an additive effect (Eye size ~ Forewing length + Family)				
Intercept	-0.1531	0.1708	-0.8967	0.3699
Forewing length	0.5182	0.1055	4.9113	<0.001
Family-Lycaenidae	-0.3298	0.1779	-1.8542	0.0637
Family-Nymphalidae	-0.0855	0.1640	-0.5210	0.6024
Family-Papilionidae	0.1455	0.2144	0.6788	0.4972
Family-Pieridae	-0.1743	0.1714	-1.0172	0.3091
Family and sex as an additive effect (Eye size ~ Forewing length + Family + Sex)				
Intercept	-0.6508	0.1262	-5.1575	<0.001
Forewing length	0.9294	0.0732	12.6988	<0.001
Family-Lycaenidae	-0.3641	0.1366	-2.6647	0.0077
Family-Nymphalidae	-0.2044	0.1257	-1.6262	0.1039
Family-Papilionidae	-0.1027	0.1636	-0.6282	0.5299
Family-Pieridae	-0.2949	0.1313	-2.2455	0.0247
Sex-Male	0.1153	0.0043	27.0530	<0.001

Table S6: Fit of evolutionary models – BM(Brownian Motion), OU(Ornstein-Uhlenbeck), BM with trend (Brownian Motion with trend), EB (Early Burst) and non-phylogenetic white noise model – using *fitContinuous* function from the *geiger* R package. The models were fitted for the eye size residuals extracted from the PGLS (eye size ~ forewing length) with the Brownian Motion correlation structure (see Table S3). 500 optimizations were used to determine the model convergence.

Model	No. of free parameters	log likelihood	AICc	AIC weights	No. of iterations with same best fit	Frequency of best fit
BM	2	71.30	-138.39	0.4956	500	1
OU	3	71.30	-136.17	0.1632	329	0.658
BM with trend	3	71.34	-136.25	0.1697	22	0.044
EB	3	71.55	-136.27	0.1715	74	0.148
White noise	2	28.43	-52.65	0	500	1

Table S7: Fit of the PGLS models to test the effect of habitat (quantified as tree cover density) on relative and absolute eye size. The models were supplied with different correlation structures and the best model was chosen based on the AIC score. Trait values (eye size and forewing length) were log10 transformed and the predictors (forewing length and tree cover density) were standardized (mean=0, SD=1) before fitting the models.

Model types	Correlation structure	DF	Log likelihod	AIC
Eye size ~ Forewing length + Habitat				
Relative eye size	Brownian Motion	4	72.9397	-137.8794
Relative eye size	Ornstein-Uhlenbeck	5	28.4079	-46.8158
Relative eye size	Pagel's lambda	5	72.9633	-135.9265
Relative eye size	OLS (Pagel's lambda=0)	4	28.4079	-48.8158
Eye size ~ Habitat				
Absolute eye size	Brownian Motion	3	32.5530	-59.1060
Absolute eye size	Ornstein-Uhlenbeck	4	3.8271	0.3458
Absolute eye size	Pagel lambda	4	32.8241	-57.6482
Absolute eye size	OLS (Pagel's lambda=0)	3	3.8271	-1.6542

Table S8: Estimates from PGLS with the Brownian Motion correlation structure (see Table S7) to test the effect of habitat (quantified as tree cover density) on the relative and absolute eye size.

Predictors	Estimate (95% CI)	Std. error	P value
Model: eye size ~ forewing length + Habitat			
Intercept	0.405 (0.31190, 0.49816)		<0.001
Forewing length	0.1933 (0.1642, 0.2225)	0.0149	<0.001
Tree cover density	-0.0116 (-0.0244, 0.0012)	0.0065	0.0805
Model: eye size ~ Habitat			
Intercept	0.4294 (0.2422, 0.6167)	0.0956	<0.001
Tree cover density	-0.0016 (-0.0272, 0.0240)	0.0131	0.9044

Table S9: Fitting linear regressions to test the effect of habitat (quantified as tree cover density) on community-weighted means of absolute and relative eye size and forewing length.

Term	Estimate	Conf. low	Conf. high	Std. error	Statistic	P value
Absolute eye size ~ Habitat						
Intercept	0.3745	0.3663	0.3828	0.0042	89.1852	<0.001
Tree cover density	-0.0003	-0.0005	-0.0001	0.0001	-2.8944	0.0039
Eye size residuals ~ Habitat						
Intercept	-0.0473	-0.0520	-0.0426	0.0024	-19.7726	<0.001
Tree cover density	-0.0003	-0.0004	-0.0002	0.0001	-4.6158	<0.001
Forewing length ~ Habitat						
Intercept	1.2992	1.2940	1.3044	0.0026	490.8167	<0.001
Tree cover density	0.0000	-0.0001	0.0001	0.0001	-0.3321	0.7398

Table S10: Fit of the PGLS models to test the effect of habitat (quantified as tree cover density) on relative and absolute eye size only for the Nymphalidae family. The models were supplied with different correlation structures and the best model was chosen based on the AIC score. Trait values (eye size and forewing length) were log10 transformed and the predictors (forewing length and canopy cover) were standardized (mean=0, SD=1) before fitting the models.

Model types	Correlation structure	DF	Log likelihood	AIC
Eye size ~ Forewing length + Habitat				
Relative eye size	Brownian Motion	4	44.9538	-81.9077
Relative eye size	Ornstein-Uhlenbeck	5	26.6355	-43.271
Relative eye size	Pagel’s lambda	5	45.0588	-80.1176
Relative eye size	OLS (Pagel’s lambda=0)	4	26.6355	-45.271
Eye size ~ Habitat				
Absolute eye size	Brownian Motion	3	19.1664	-32.3328
Absolute eye size	Ornstein-Uhlenbeck	4	6.5651	-5.1301
Absolute eye size	Pagel’s lambda	4	19.1665	-30.3331
Absolute eye size	OLS (Pagel’s lambda=0)	3	6.5651	-7.1301

Table S11: Estimates from PGLS with the Brownian Motion correlation structure (see Table S10) to test the effect of habitat (quantified as tree cover density) on the relative and absolute eye size only for the Nymphalidae family.

Predictors	Estimate (95% CI)	Std. error	P value
Model: eye size ~ forewing length + Habitat			
Intercept	0.3745 (0.2732, 0.4758)	0.0517	<0.001
Forewing length	0.2035 (0.1671, 0.2399)	0.0186	<0.001
Tree cover density	-0.0105 (-0.0245, 0.0034)	0.0071	0.1495
Model: eye size ~ Habitat			
Intercept	0.4615 (0.2356, 6874)	0.1153	0.0004
Tree cover density	0.0072 (-0.0234, 0.0378)	0.0156	0.6476

Table S12: Fitting linear regressions to test the effect of tree cover density on community-weighted means of absolute and relative eye size and forewing length only for the Nymphalidae family.

Term	Estimate	Conf. low	Conf. high	Std. error	P value
Absolute eye size ~ Habitat					
Intercept	0.4298	0.42138	0.43817	0.0043	<0.001
Tree cover density	0.0002	-0.00003	0.00037	0.0001	0.1011
Eye size residuals ~ Habitat					
Intercept	-0.0292	-0.03412	-0.0242	0.0025	<0.001
Tree cover density	0.0001	-0.00002	0.0002	0.0001	0.1207
Forewing length ~ Habitat					
Intercept	1.3472	1.34339	1.35092	0.0019	<0.001
Tree cover density	0.0001	-0.00004	0.00014	0.0000	0.2389

Processing and segmentation of 3D scans and measuring 3D eye surface area

We processed the 3D scans (as a stack of 16-bit .tiff files) in 3D Slicer program ver 5.0.3 and 5.4.0 (https://www.slicer.org/) using additional functionalities from the SlicerMorph) plugin. Below, we provide details of the workflow used for segmentation of the structure of interest (i.e. eyes) and measuring eye surface area.

1. *Importing image stacks*

Image stacks were imported using the 'ImageStacks' module from the SlicerMorph plugin. The voxel size of the scans was provided at this stage (in mm). Figure S1A (left) shows different views of the raw 3D images representing a butterfly head.

2. *Segmentation of the head*

Segmentation of the head from the raw 3D image was carried out using the functionalities from the *'Segment Editor'* module. Specifically, the *Threshold* function was used such that the outline of the head could be clearly demarcated. Since we are only interested in eyes, body parts which are not of interest (e.g. proboscis, parts of thorax, antennae) were removed using *Scissors*. Additional cleaning was carried out using the *Islands* function in this module. At this stage, if there were holes in the 3D model (which was often the case), they were filled using the *Paint* function by editing in the 3D view. Figure S1A (right) shows the outline of the head after carrying out segmentation and the 3D model of the head.

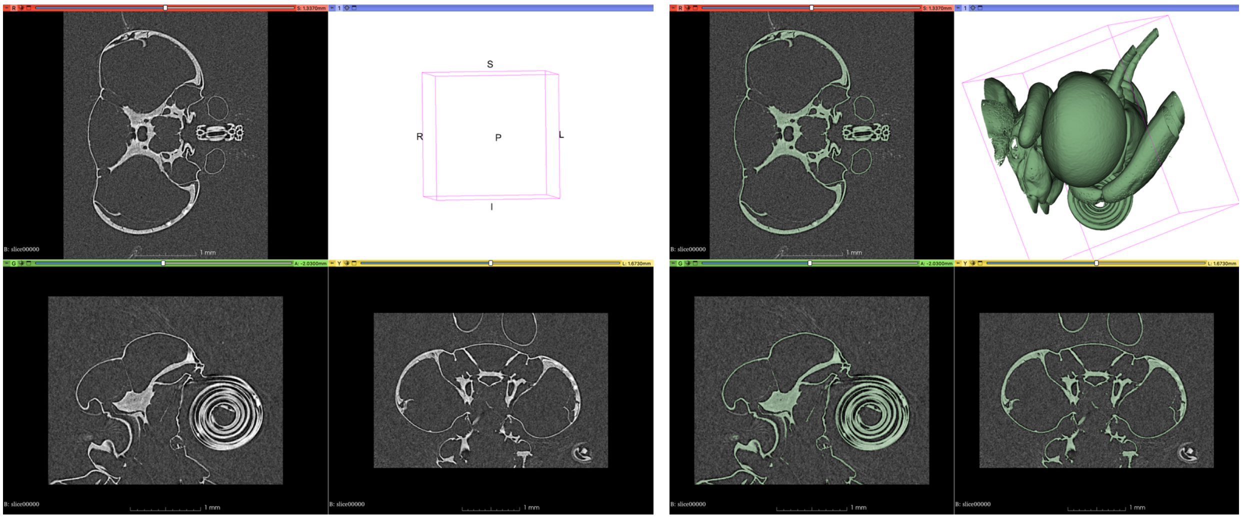

Figure S1A. The left panel shows different views of the scan of the butterfly head after importing using the 'ImageStacks' module. The right panel shows the same views as the left panel, but after applying the threshold to demarcate the outline of the head and then reconstructing the 3D model of the head.

3. *Demarcating the boundary of the cornea or eye surface*

Since it was not possible to segment only the cornea from the head model (Figure S1B, left), as these are dried museum specimens and the density of dried cuticle is almost similar across the body. Thus, to be able to segment only the cornea, landmarks were placed around the boundary of the cornea (Figure S1B, middle) using the *Closed Curve* from the 'Markups' module. Before this step, the segmentation was converted to a 3D model ('Data' module --> right click on the segmentation of interest --> choose 'convert to model'; Figure S1B, left). After placing the curve (Fig S1B, middle), we defined settings for the curve from the *Curve Settings* option in the 'Markups' module. The following settings were chosen: curve type = spline, constrain to model = the head model that we created. At this stage, we also resampled the number of points on the curve (usually 30) from the *Resample* option in the 'Markups' module.

Note that, placing the curve on the model is the most time-consuming part and also error-prone. To ensure that the points were placed as accurately as possible on the corneal boundary, we frequently checked the position of our points by toggling between the different 2D and 3D views and adjusted the points by dragging them as necessary. Measurement error arising from this step, both within and across individuals, was the motivation behind measuring repeatability (see Methods section for details).

Finally, after placing the boundary, we performed *Curve Cut* from the ‘Dynamic Modeller’ module. This function creates the cast or replica of the inside model surface demarcated by the curve. The cast or the surface of the outer eye surface is shown in Figure S1B (right). The surface area of this model was measured from the *Models* module.

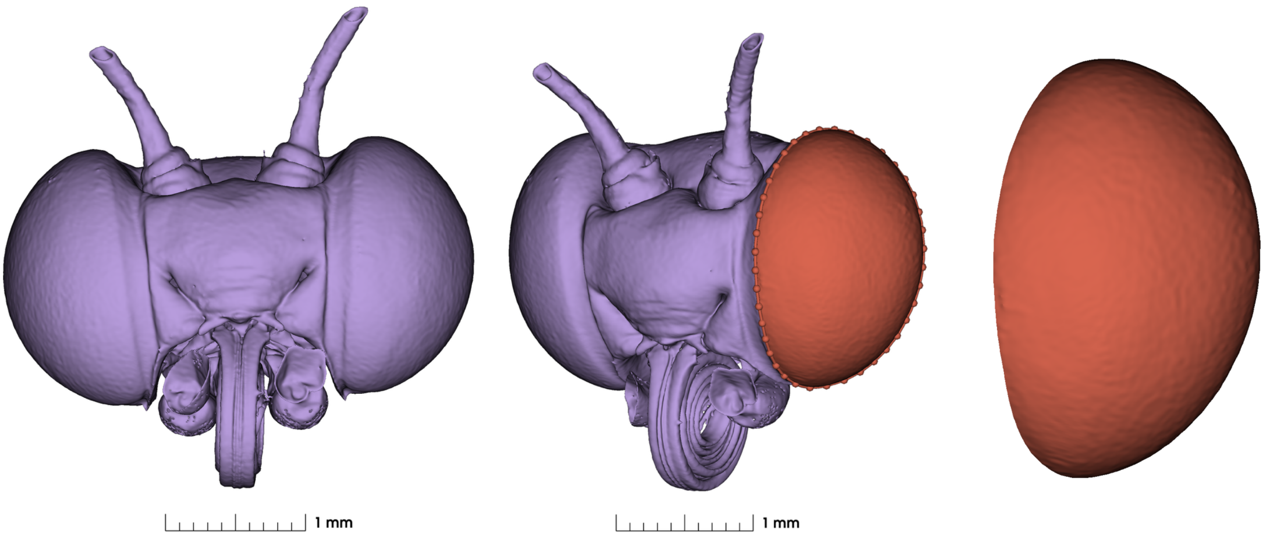

Figure S1B. The images show the 3D reconstruction of the entire head (left), the placement of the curve and demarcation of the cornea (middle) and the model of the eye surface area only (C) after performing the curve cut.

Measuring wing traits from 2D images

We measured the wing traits (forewing length and forewing area, as shown below) of the pinned museum specimens. For forewing length, the landmark was placed on the hinge where the wing first joins the thorax and tip of the forewing, usually following a vein (Fig. S2). Forewing area was measured as an area enclosed by three clear landmarks (Fig. S2). Note that since we are measuring species spanning large divergence times, wing venation can vary across families and sometimes even across species within a family. Thus, our landmarks are not exactly homologous. However, such minor variation in wing venation, which could slightly affect the forewing length and area, is not expected to exceed the variation across species. Additionally, we measured repeatability by measuring these wing traits twice by two individuals and found them highly repeatable (R^2=0.99, see Fig. S4). Also, note that we carried out all comparative analyses using forewing length as a proxy for body size and there was a strong correlation between forewing length and forewing area (R2=0.98, Fig. S3).

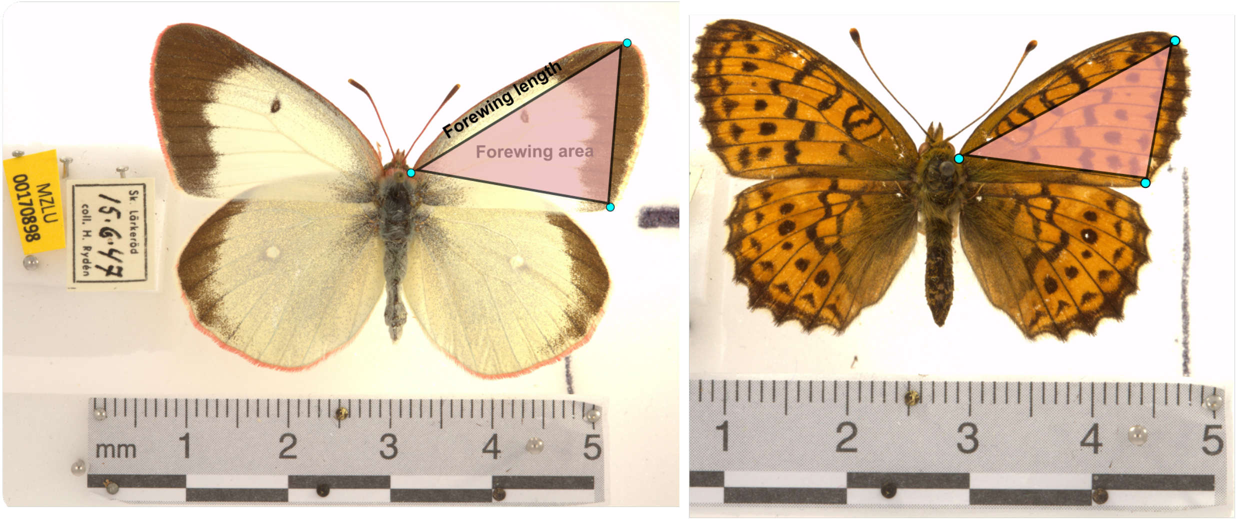

Figure S2: Measuring wing traits (forewing length & forewing area) of a pinned museum specimens: *Colias palaeno* (left) and *Boloria ino* (right).

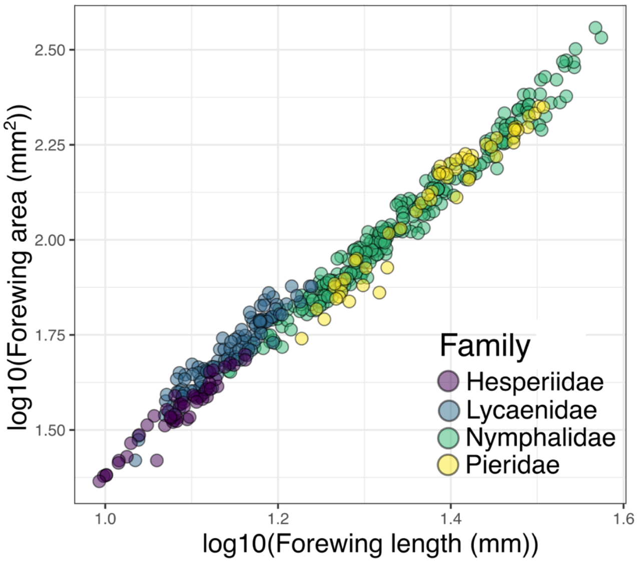

Figure S3: Correlation between log10 forewing length and forewing area (R^2=0.98). Forewing length was used as a body size proxy for all the comparative analyses.

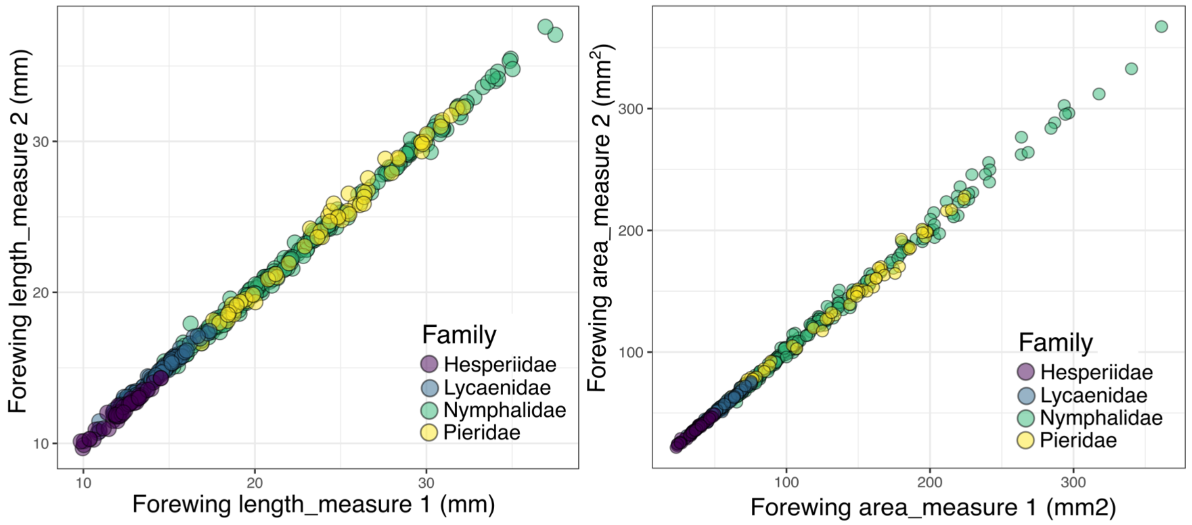

Figure S4: Measuring repeatability of wing traits: forewing length (left) and forewing area (right). Overall repeatability was high for both wing traits (R^2=0.99).

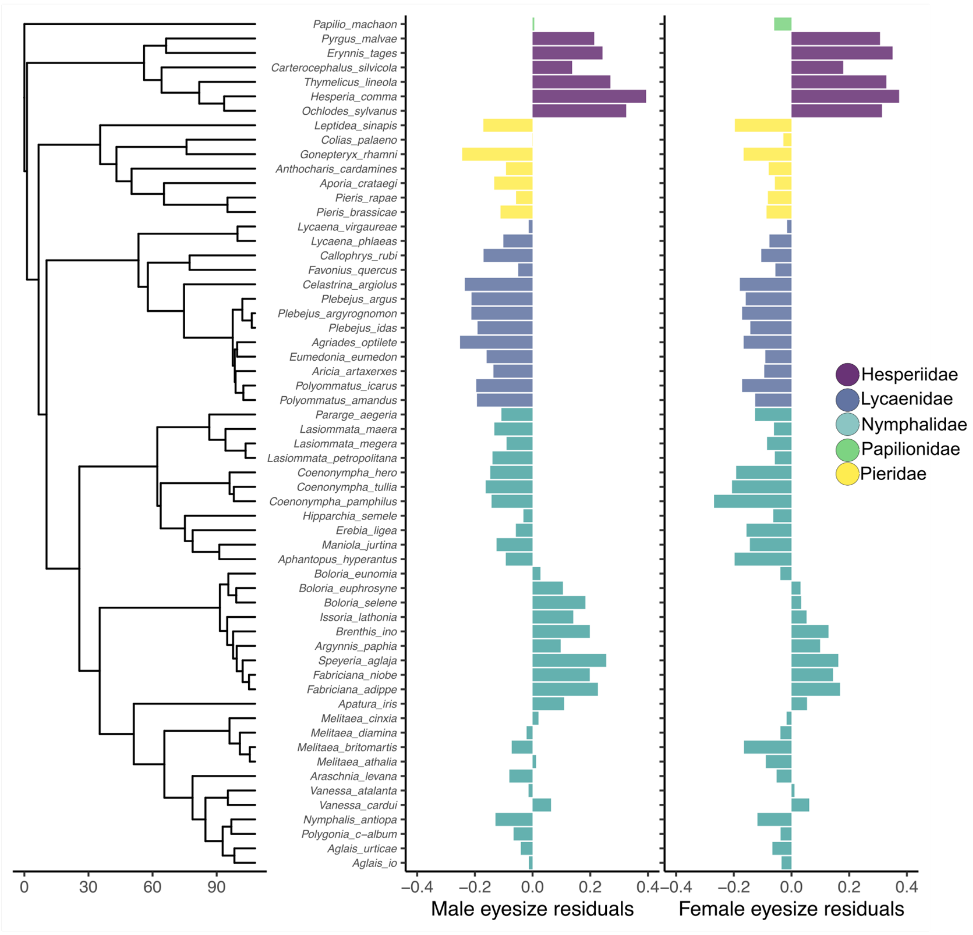

Figure S5: Depicting eye size residuals of males and females from PGLS (eye size ~ forewing length) fitted seperately for sexes. PGLS (with the Brownian Motion correlation structure) were fitted on the pooled species-averaged data according to sexes. Bars are colored according to the butterfly families (legend on the right).

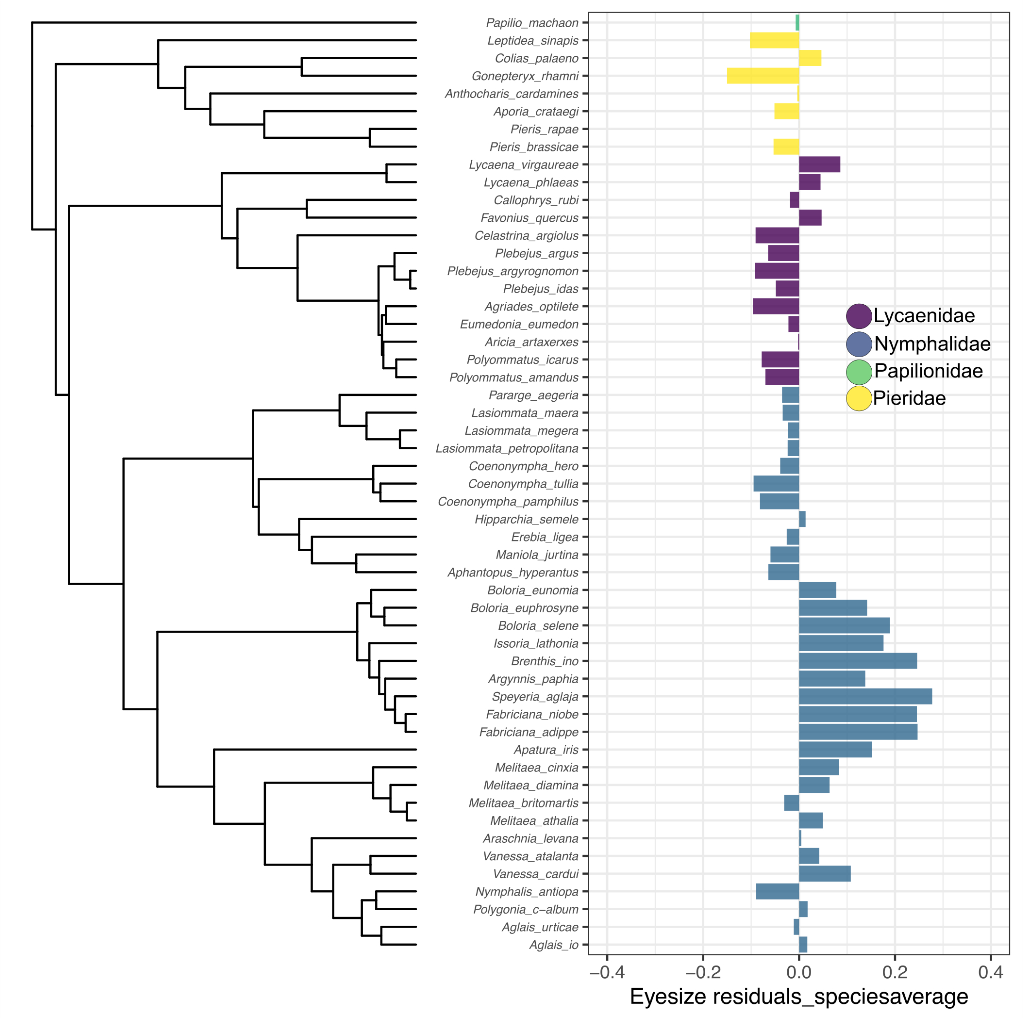

Figure S6: Depicting eye size residuals of males and females from PGLS (eye size ~ forewing length) fitted on the pooled species-averaged data (with the Brownian Motion correlation structure) after removing species from the Hesperiidae family.

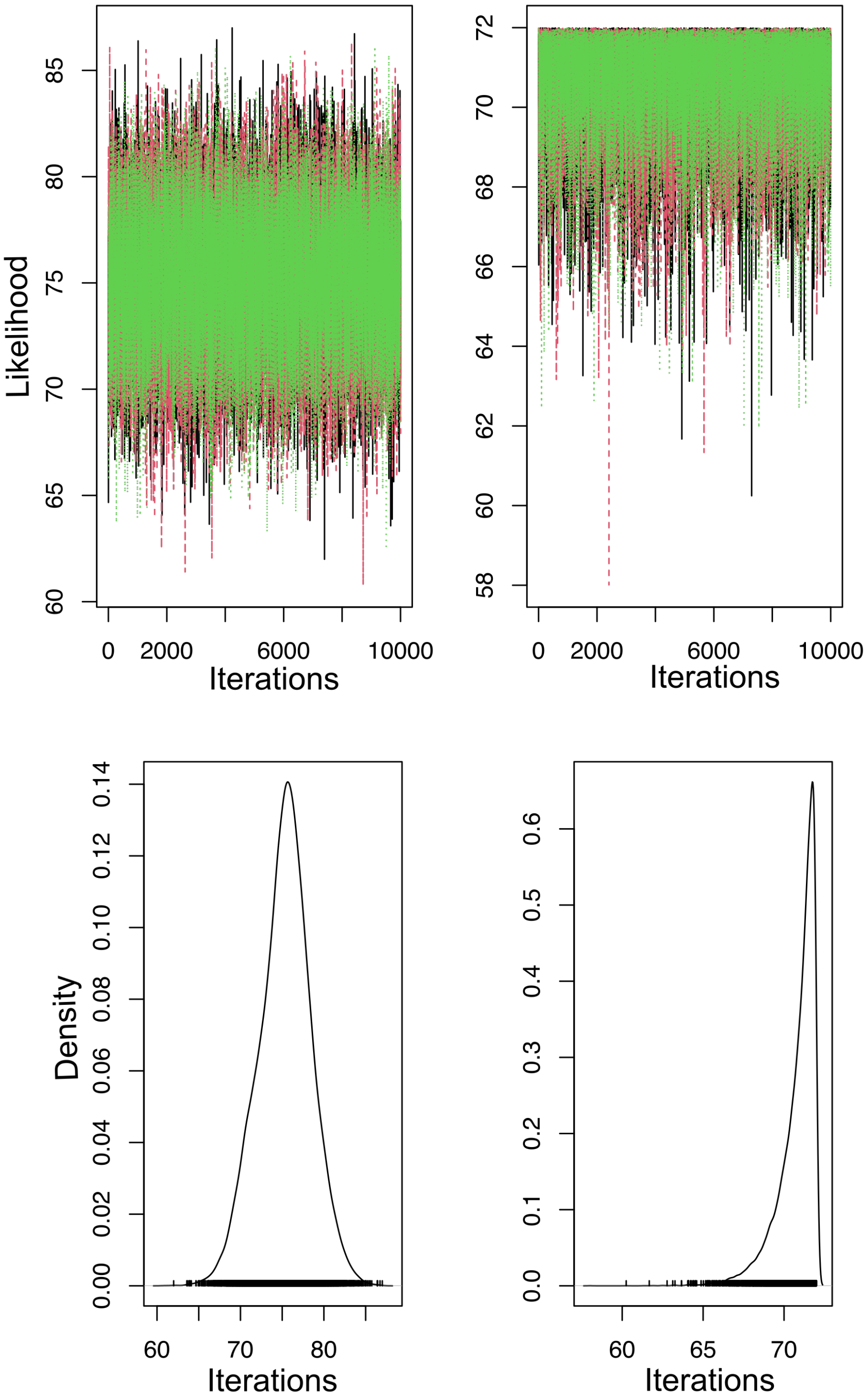

Figure S7: Trace plot (top) and distribution of likelihood values (below) across three different independent runs of the Bayesian variable-rates and homogenous-rates model. See Table S3 for effective sample size and Gelman-Rubin estimates.

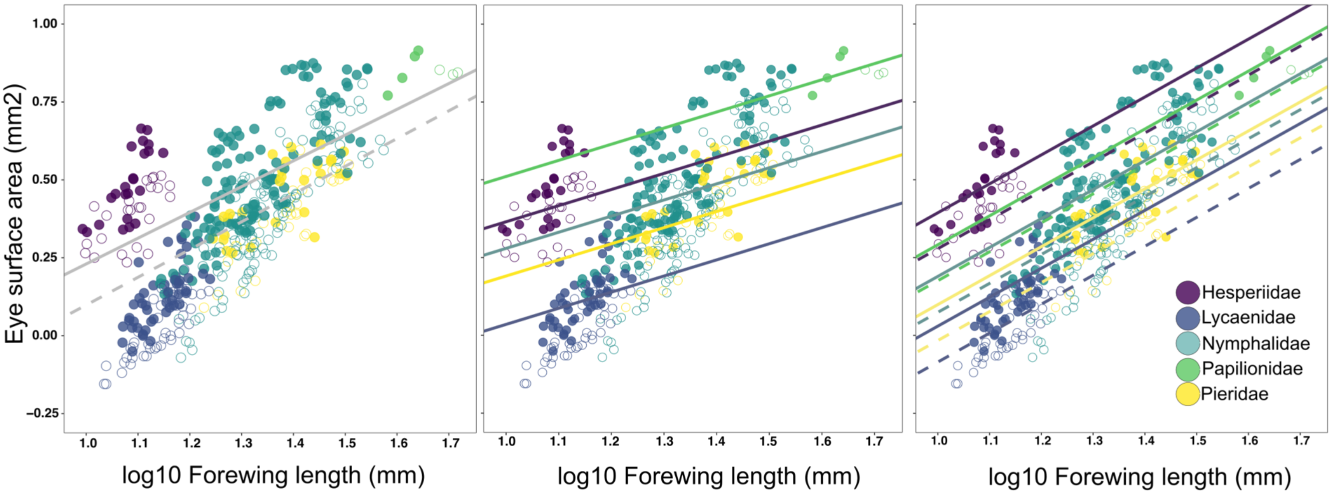

Figure S8: Allometry between eye size and forewing length fitted using the *pglmm* function (from *phyr* package) on the entire data. In the left plot, Sex was included as an interactive effect with forewing length (model: eye size ~ Forewing length*Sex). In the middle plot, Family was included as an additive effect (model: eye size ~ Family) and in the right plot, both Family and Sex were added as an additive effect (model: eye size ~ forewing length + family + sex). Solid and dashed lines in both plots indicate male and female regression lines, respectively. Similarly, filled and open circles indicate males and females, respectively. Points and lines in the middle and right plot are colored according to the families (legend provided as an inset in the right plot).

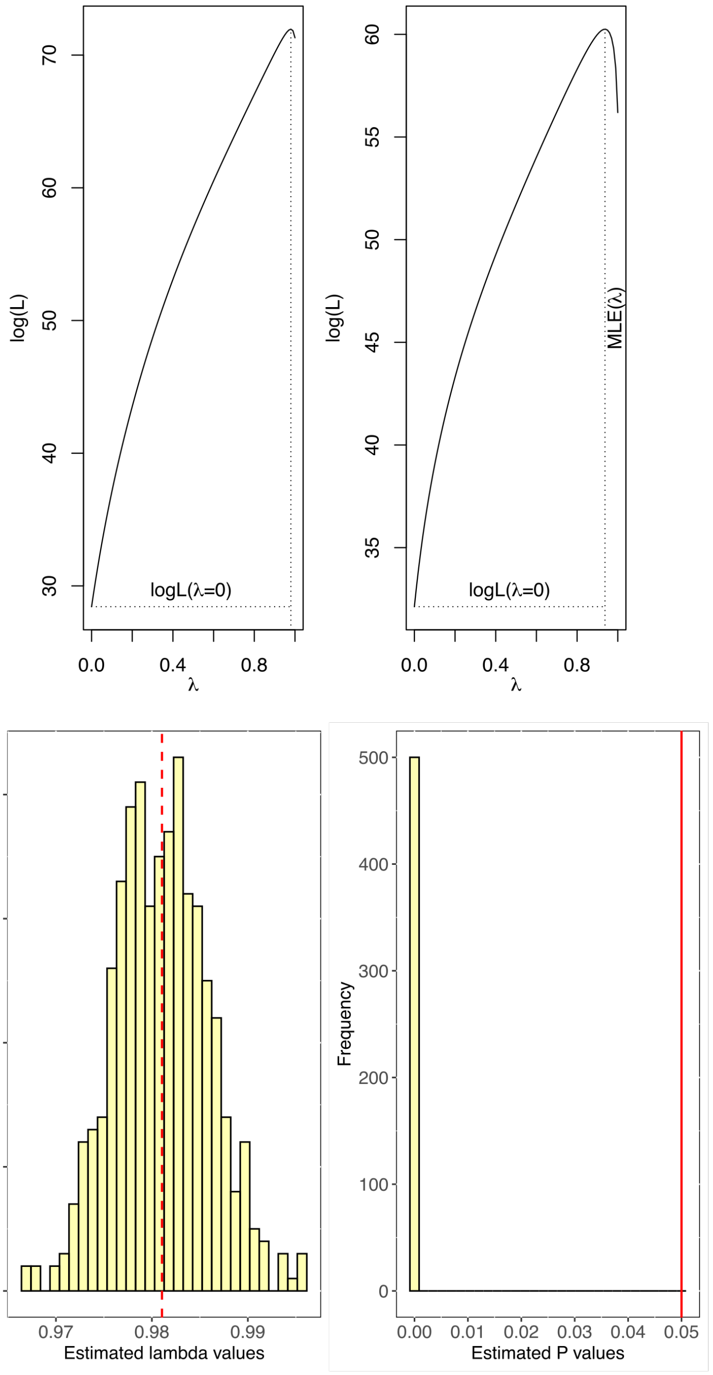

Figure S9: Maximum likelihood profiles of Pagel's lambda estimation for eye size residuals (top left) and forewing length (top right) and the estimates of lambda values across 500 posterior trees and estimated P values for eye size residuals (bottom panel).

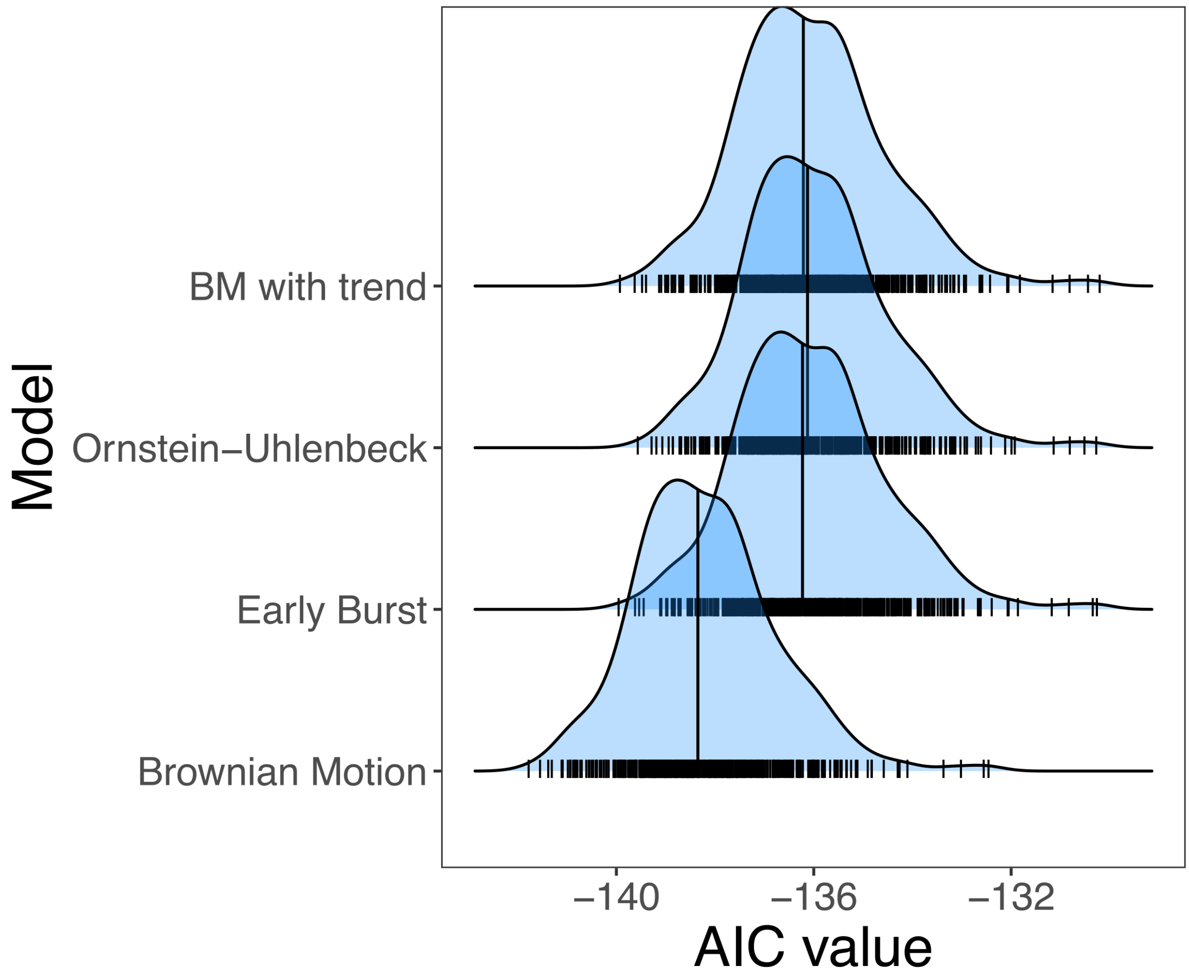

Figure S10: AIC scores showing the fit of species-averaged eye size residuals to several models of continuous trait evolution (fitted using *fitContinuous* function from *geiger* R package). The non-phylogenetic white model was also fitted but excluded from the plot due to very high AIC values, thus, affecting the scale of the axis. On average, the AIC value of the white noise model was -52.

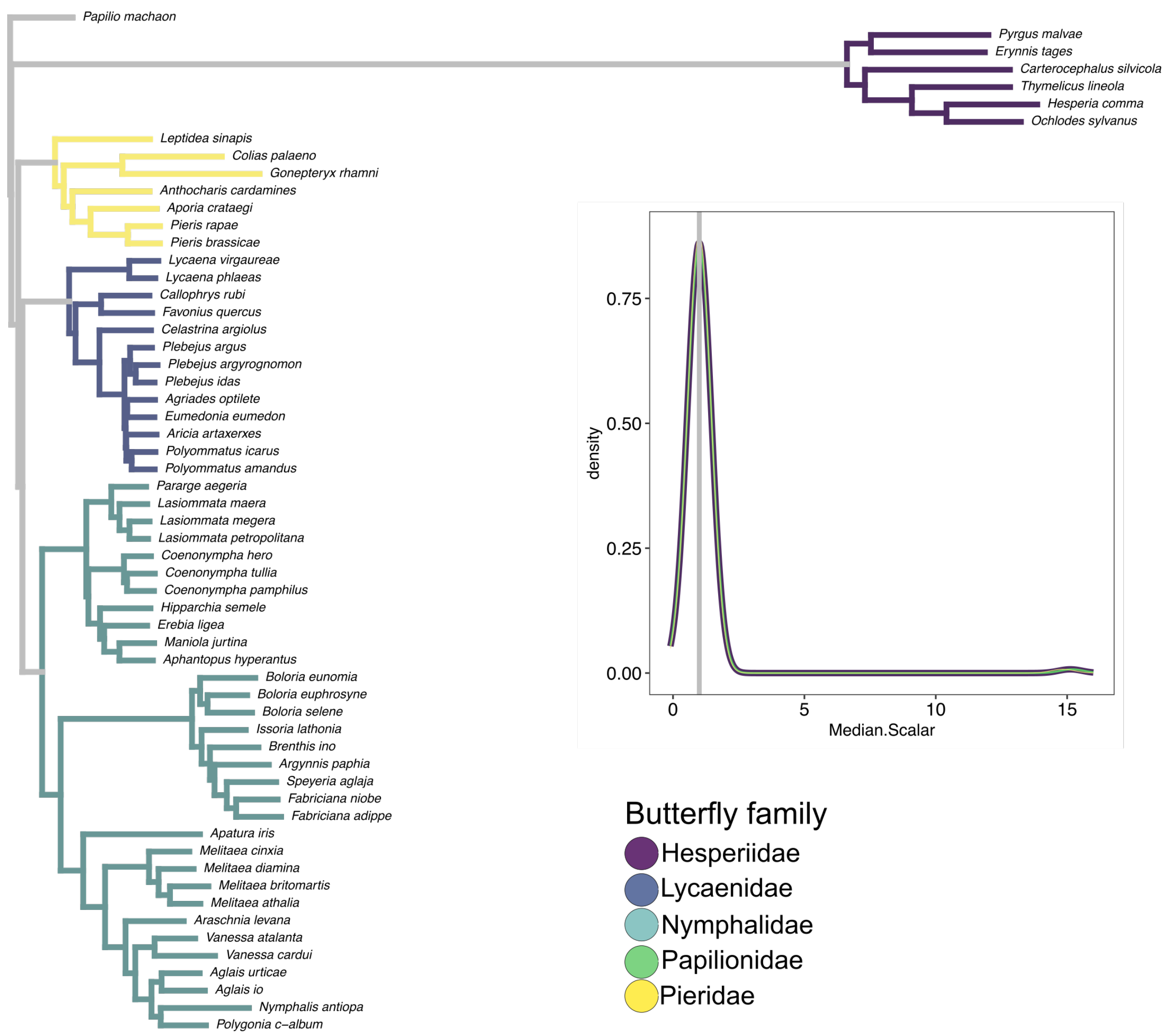

Figure S11: Consensus tree obtained from the Bayesian variable-rate model used for quantifying rate heterogeneity in the eye size evolution. The branches/clades are stretched in proportion to the rate scalar (*r*), with *r*>1 and *r*<1 indicating higher and lower rates, respectively, while *r*=1 indicates rates equal to the homogenous Brownian Motion rate. The tree shows that the Hesperiidae clade has the highest rate and hence has been stretched the most. The clade of fritillary butterflies has been stretched only slightly, with r slightly exceeding 1. Colors of clades correspond to butterfly families. The density plot shows the posterior distribution of median rate scalar (*r*) for three independent runs (note that the lines are overlapping), with majority of rate scalars equal to 1, suggesting overall rate heterogeneity is low.

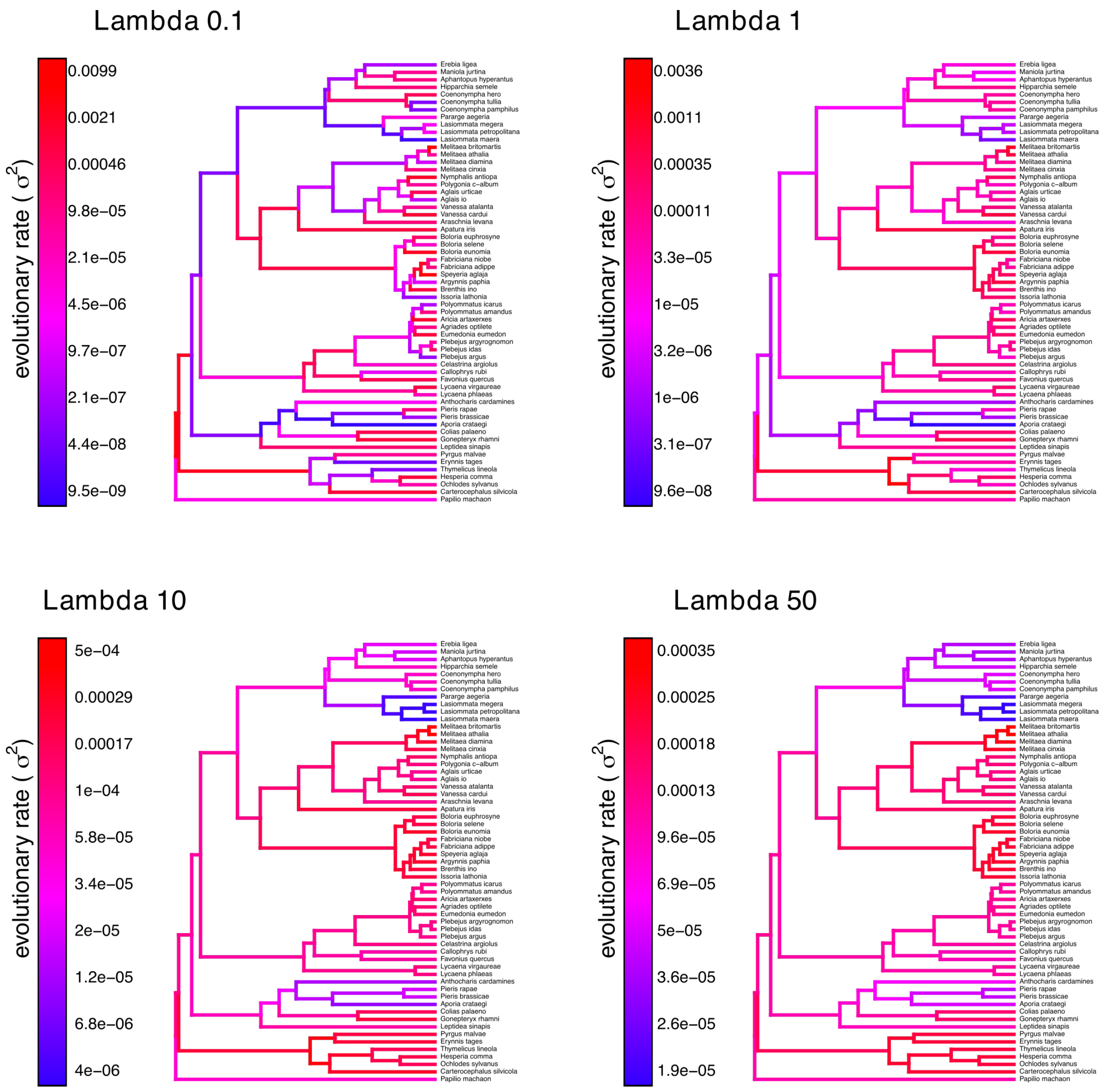

Figure S12: Quantifying heterogeneity in evolutionary rates using the penalized-likelihood based variable-rates model. Low lambda values allow dramatic changes in evolutionary rates with lower penalty, while higher lambda values imposes higher penalty and reduces too drastic changes in evolutionary rates.

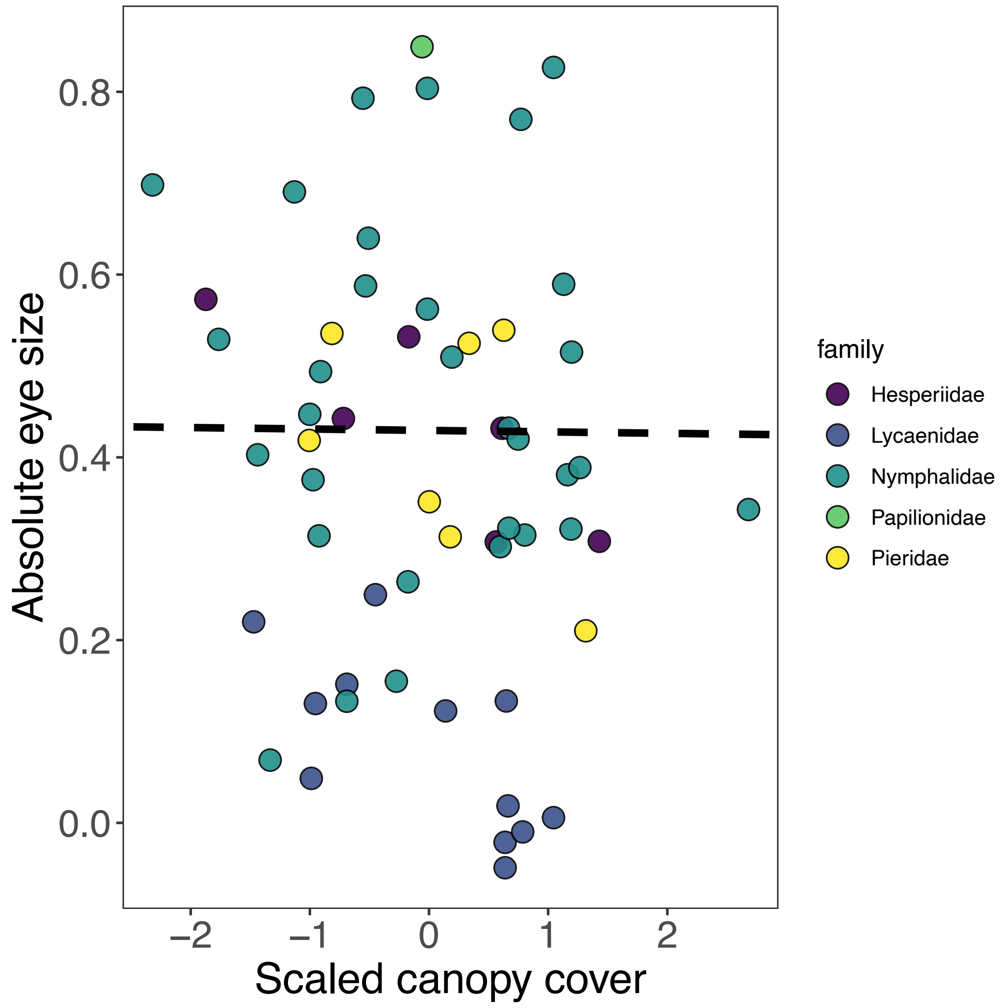

Figure S13: PGLS between tree cover density (standardized to have mean of 0 and SD of 1) and species-averaged absolute eye size. Regression estimates are presented in Table S8. Points are colored according to the butterfly families.

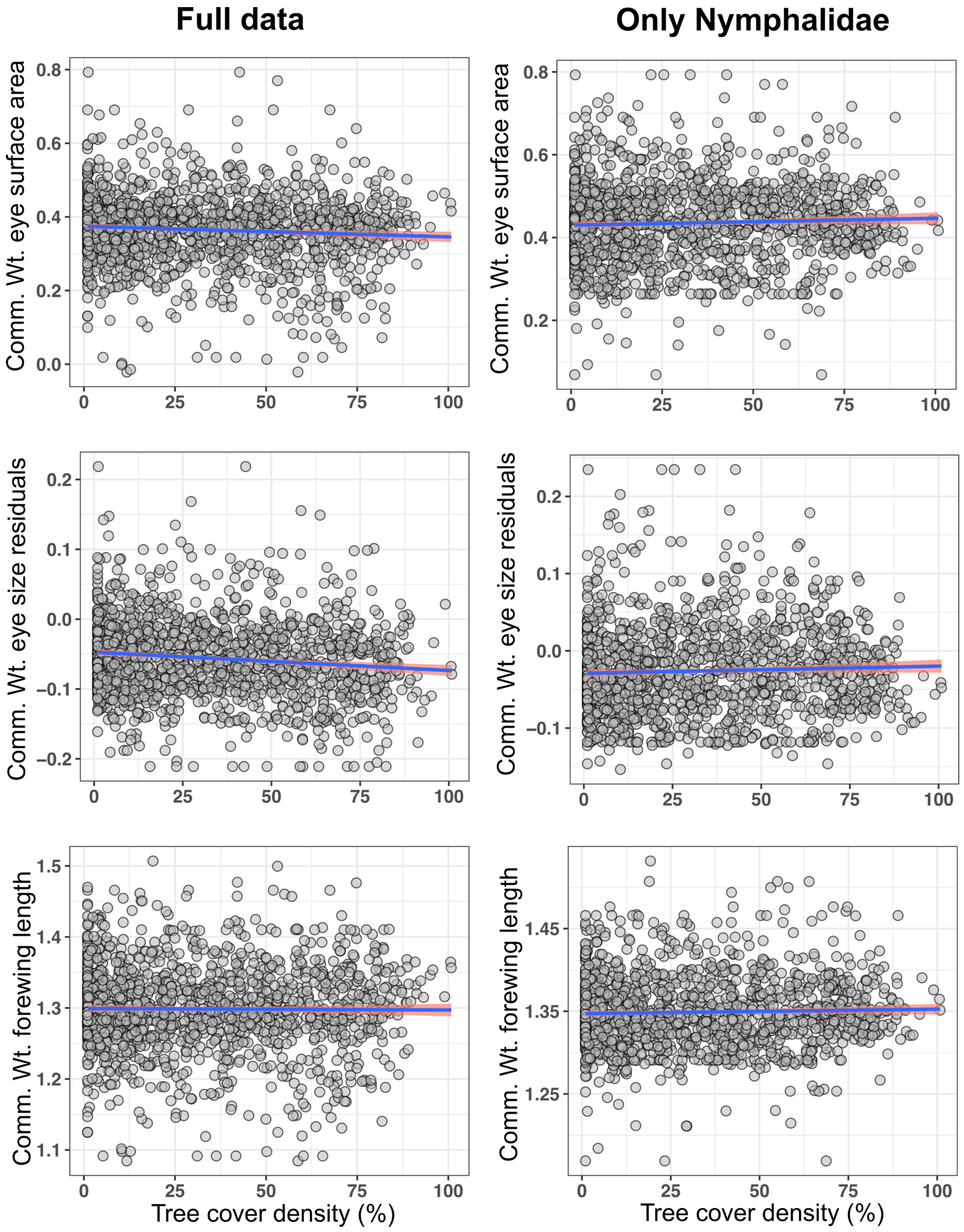

Figure S14: Linear regression between tree cover density and community-weighted means of relative and absolute eye size and forewing length for the entire data and only for the Nymphalidae family.

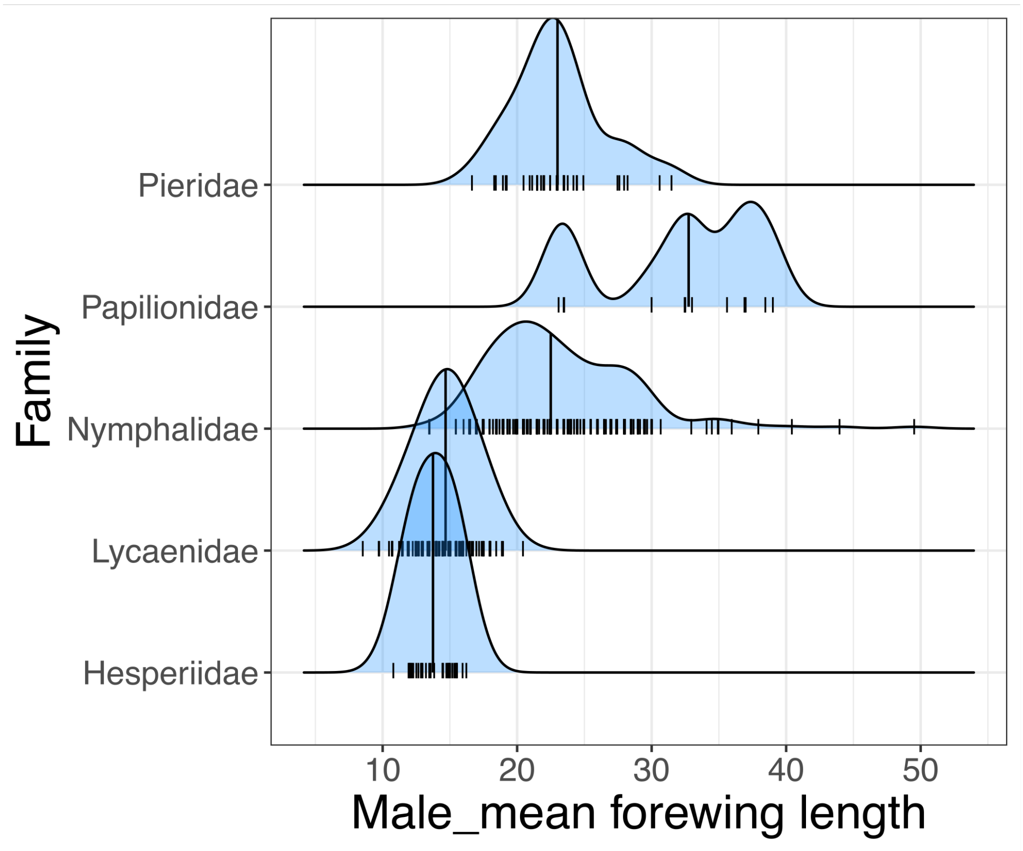

Figure S15: Distribution of forewing length across males (left) and females (right) across 363 European butterflies. Data for forewing length was obtained from Middleton-Welling et al. (2020). The vertical line on each density curve represents sthe median forewing length.

Middleton-Welling, J., *et al*. (2020). A new comprehensive trait database of European and Maghreb butterflies, Papilionoidea. *Scientific Data*, 7(1), 351.
